# Mimiviridae: clusters of orthologous genes, reconstruction of gene repertoire evolution and proposed expansion of the giant virus family

**DOI:** 10.1186/1743-422X-10-106

**Published:** 2013-04-04

**Authors:** Natalya Yutin, Philippe Colson, Didier Raoult, Eugene V Koonin

**Affiliations:** 1 National Center for Biotechnology Information, National Library of Medicine, National Institutes of Health, Bethesda, MD 20894, USA; 2 URMITE, UM 63 CNRS 7278 IRD 198 INSERM U1095, Faculté de Médecine, Aix-Marseille University, 27 Boulevard Jean Moulin, Marseille Cedex 5, 13385, France

## Abstract

**Background:**

The family *Mimiviridae* belongs to the large monophyletic group of Nucleo-Cytoplasmic Large DNA Viruses (NCLDV; proposed order Megavirales) and encompasses giant viruses infecting amoeba and probably other unicellular eukaryotes. The recent discovery of the *Cafeteria roenbergensis* virus (CroV), a distant relative of the prototype mimiviruses, led to a substantial expansion of the genetic variance within the family *Mimiviridae*. In the light of these findings, a reassessment of the relationships between the mimiviruses and other NCLDV and reconstruction of the evolution of giant virus genomes emerge as interesting and timely goals.

**Results:**

Database searches for the protein sequences encoded in the genomes of several viruses originally classified as members of the family *Phycodnaviridae*, in particular Organic Lake phycodnaviruses and *Phaeocystis globosa* viruses (OLPG), revealed a greater number of highly similar homologs in members of the *Mimiviridae* than in phycodnaviruses. We constructed a collection of 898 Clusters of Orthologous Genes for the putative expanded family *Mimiviridae* (MimiCOGs) and used these clusters for a comprehensive phylogenetic analysis of the genes that are conserved in most of the NCLDV. The topologies of the phylogenetic trees for these conserved viral genes strongly support the monophyly of the OLPG and the mimiviruses. The same tree topology was obtained by analysis of the phyletic patterns of conserved viral genes. We further employed the mimiCOGs to obtain a maximum likelihood reconstruction of the history of genes losses and gains among the giant viruses. The results reveal massive gene gain in the mimivirus branch and modest gene gain in the OLPG branch.

**Conclusions:**

These phylogenomic results reported here suggest a substantial expansion of the family *Mimiviridae*. The proposed expanded family encompasses a greater diversity of viruses including a group of viruses with much smaller genomes than those of the original members of the *Mimiviridae*. If the OLPG group is included in an expanded family *Mimiviridae*, it becomes the only family of giant viruses currently shown to host virophages. The mimiCOGs are expected to become a key resource for phylogenomics of giant viruses.

## Background

The Nucleo-Cytoplasmic Large DNA Viruses (NCLDV) comprise a major, apparently monophyletic group of viruses that consists of 6 established virus families and a 7th putative family [1-3]. The NCLDV infect animals and diverse unicellular eukaryotes and either replicate exclusively within the so-called virus factories in the cytoplasm of the host cells [4,5], or go through both cytoplasmic and nuclear stages in their reproduction cycle [6].

With the exception of some viruses in the Phycodnaviridae family that do not encode their own RNA polymerase subunits and hence depend on the host for transcription, the NCLDV do not show strong dependence on the host replication or transcription systems for completing their replication [6,7]. This relative independence of the NCLDV from the host cells is consistent with the fact that these viruses encode many conserved proteins that mediate most of the processes essential for viral reproduction. These key proteins include DNA polymerases, primases, helicases, flap nucleases and DNA clamps that are responsible for DNA replication; Holliday junction resolvases and topoisomerases involved in genome DNA manipulation and processing; transcription factors that function in transcription initiation and elongation; ATPase pumps for DNA packaging; chaperones involved in the capsid assembly and the capsid proteins themselves [1-3,8]. Although only 5 genes are conserved in all NCLDV (with sequenced genomes), evolutionary reconstruction using maximum parsimony or maximum likelihood approaches mapped between 40 and 50 genes to the putative common ancestor of the NCLDV [2]. Given the compelling evidence in favor of the monophyly of the NCLDV, it has been recently proposed to formally recognize this group of viruses as a new taxon, the order *Megavirales*[9].

The best characterized family of the NCLDV is the Poxviridae that includes numerous viruses infecting animals including smallpox virus, the causative agent of one the most devastating human infectious diseases, and vaccinia virus, a classic model of molecular virology [10]. Recently, however, the group of the NCLDV that had attracted the most attention had been the family *Mimiviridae* that encompasses by far the largest known viruses [11-13]. The giant Mimivirus, the prototype of the family, was isolated from *Acanthamoeba polyphaga* and shown to possess ~1.2 Mb genome and encompass more than 1000 protein-coding genes [14]. Subsequently, 3 more genomes of related viruses have been sequenced, 2 of these even slightly larger than the Mimivirus genome [11,15-19]. In addition, approximately 20 mimiviruses have been detected through genomic and proteomic surveys but have not yet been characterized in detail [20]. Most of the currently identified mimiviruses infect the freshwater protist (and an opportunistic human pathogen) *Acanthamoeba* but the current genome size record holder, *Megavirus chiliensis*, was isolated from ocean water although its specific host remains unknown [21]. Recently a giant (albeit somewhat smaller than the previously isolated mimiviruses, with a 700 Kb genome) virus has been isolated from the marine flagellate *Cafeteria roenbergensis* (and accordingly designated CroV after *Cafeteria roenbegensis* virus) [22,23]. Phylogenetic analysis of the core NCLDV genes indicated that, among the other NCLDV, CroV was the closest relative of the mimiviruses and could be classified as a distant member of the family *Mimiviridae*[22,24]. Furthermore, numerous sequences homologous to mimivirus genes have been identified in marine metagenomic samples indicating that mimiviruses are common in these habitats [25,26]. Taken together, these findings indicate that *Mimiviridae* is an expansive family of giant viruses the true diversity of which remains largely untapped.

In addition to all the core NCLDV genes, members of the family *Mimiviridae* possess many genes the presence of which in viruses is unexpected, in particular genes encoding components of the translation systems such as aminoacyl-tRNA synthetases and translation factors [14,21]. The discovery of these genes that comprise parts of the core molecular machinery of all cellular life forms but are uncharacteristic of viruses fueled the debate on the controversial possibility that mimiviruses represent a “fourth domain of life” [9,14,24,27-29].

A notable feature of giant viruses is that they harbor their own mobilome, a collection of diverse selfish elements that depend on a giant virus for their reproduction. In addition to self-splicing introns and inteins, mimiviruses support the replication of transpovirons, a distinct type of linear plasmids, and virophages, small viruses that replicate within the intracellular factories of the host giant virus [30,31]. The first discovered virophage, dubbed Sputnik, is a parasite of the Mamavirus and closely related mimiviruses, and is an icosahedral virus with an approximately 20 kilobase dsDNA genome [16]. Subsequently, it has been shown that Sputnik can integrate into the genome of the host mimiviruses [30]. Two distinct virophages have been shown to infect CroV [32] and Organic Lake phycodnavirus [33]; these virophages resemble Sputnik in terms of the overall virion and genome structure but substantially differ in their gene repertoires.

As part of an effort to understand the evolutionary history and ultimately the origin of the giant viruses, we constructed Clusters of Mimivirus Orthologous Genes (mimiCOGs) and reassessed the relationship of the family *Mimiviridae* with the other NLCDV. The result is a potential major expansion of the family *Mimiviridae* that is shown to include several viruses previously classified as members of Phycodnaviridae.

## Results and discussion

### Comparative genomics of the putative expanded family Mimiviridae

**Figure 1:**
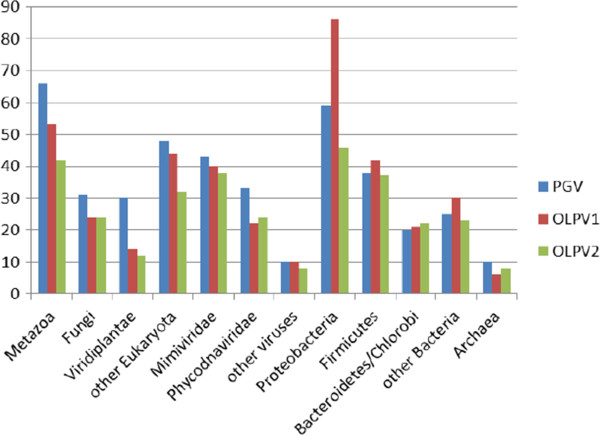
**Phyletic distribution of Refseq best BLAST hits of *P. globosa *virus 12 T (PGV), Organic Lake phycodnavirus 1 (OLPV1), and Organic Lake phycodnavirus 2 (OLPV2). **The family *Mimiviridae *included *C. roenbergensis *virus BV-PW1, *A. polyphaga *mimivirus, and Megavirus chiliensis. Phycodnaviridae include: *Bathycoccus *sp. RCC1105 virus BpV1, *Ectocarpus siliculosus *virus 1, *Emiliania huxleyi *virus 86, *Feldmannia* species virus, *Micromonas *sp. RCC1109 virus MpV1, *Ostreococcus lucimarinus *virus OlV1, *O. tauri *virus 1, *O. tauri *virus 2, *O. tauri *virus OsV5, and Chloroviruses.

In the course of phylogenomic study of the NCLDV, we noticed that in sequence database searches the proteins from some large DNA viruses assigned to the family *Phycodnaviridae*, namely Organic Lake phycodnaviruses [33] and *Phaeocystis globosa* viruses 12 T and 14 T [34,35] produce a substantially greater number of best hits into mimiviruses than into phycodnaviruses (Figure 1, Additional file 1).

To further investigate the evolutionary provenance of these poorly characterized giant viruses (hereinafter OLPG, after Organic Lake and *Phaeocystis globosa* viruses), we conducted an in depth phylogenomic analysis of the previously identified and putative new members of the family *Mimiviridae*. To this end, we constructed clusters of orthologous genes (COGs [36,37]) from the genomes of 4 mimiviruses (*Acanthamoeba castellanii* mamavirus, *Acanthamoeba polyphaga* mimivirus, *Megavirus chiliensis*, and Moumouvirus), CroV, and 3 OLPG [Organic Lake phycodnavirus 1, Organic Lake phycodnavirus 2 (these two genomes are still incomplete) and *Phaeocystis globosa* virus 12 T)]. The gene products encoded in these 8 genomes were retrieved from GenBank yielding a total of 5,677 protein sequences. These viral proteins were grouped into clusters of likely orthologs using a modified COG procedure [38] (see Methods for details). Clusters were manually edited and annotated using the results of RPS-BLAST and PSI-BLAST searches for the constituent proteins (Additional file 2 and see Methods). This procedure yielded 898 clusters of candidate orthologous genes from the putative expanded family *Mimiviridae* (hereinafter mimiCOGs). The mimiCOGs then were merged into the previously constructed clusters of orthologous genes for all NCLDV (NCVOGs [8]) (see Methods for details).

**Figure 2:**
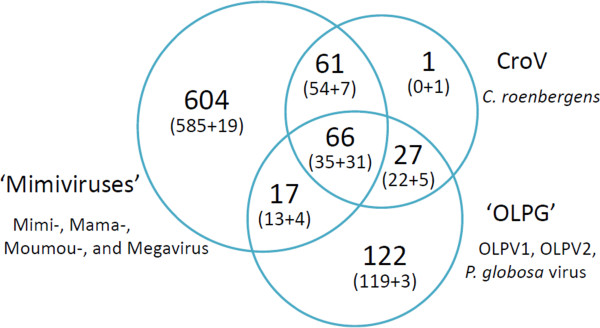
**Virus species content of the mimiCOGs. **The Venn diagram shows the numbers of mimiCOGs that are unique to and shared between three phyletic groups: *Mimiviridae*, CroV and OLPG.

Fifty-two genes are present in all 8 genomes of the members of the putative expanded family *Mimiviridae* (Table 1). In addition, 10 other genes are missing in one or two OLPG genomes but present in all genomes of the *Mimiviridae*; these genes also might be conserved in all analyzed viruses given the incompleteness of the OLPG genomes. These conserved genes include mostly the core genes with essential functions in viral replication and virion morphogenesis that are also widely represented in other NCLDV and are likely to be ancestral to this entire group of viruses [8]. However, beyond the core gene set, the genes conserved in the *Mimiviridae* and the OLPG encode several additional proteins implicated in viral replication (e.g. RNAse H, two paralogous small subunits of replication factor C and topoisomerase II) and transcription (e.g. TATA-binding protein) as well as proteins implicated in modification of host cell systems during virus infection such as a homolog of translation elongation factor 2E and ubiquitin C-terminal hydrolase (Table 1). Apart from the 52 genes that are conserved in all analyzed viral genomes, the majority of the mimiCOGs are Mimivirus-specific and missing OLPG (Figure 2). The set of OLPG-specific genes is considerably smaller. Interestingly, these genes encode several functions that have not been previously identified in viruses including the first gene for proteorhodopsin identified in virus genomes [39].

**Table 1:** **Conserved proteins of the putative extended *Mimiviridae *family**


**Proteins present in all 8 *Mimiviridae *genomes**			
CLS10031	A1L transcription factor VLTF-2	CLS10052	Proliferating cell nuclear antigen
CLS10071	A2L transcription factor VLTF-3	CLS10035	protein disulfide Isomerase/thioredoxin family
CLS10199	asnB, asparagine synthetase B	CLS10216	putative DNA-directed RNA polymerase II subunit N
CLS10039	capsid protein	CLS10047	replication factor C small subunit
CLS10262	D5-like helicase-primase	CLS10049	replication factor C small subunit
CLS10015	DEAD/SNF2-like helicase or ATP-dependent RNA helicase	CLS10258	ribonuclease H
CLS10089	DNA directed RNA polymerase subunit L	CLS10041	ribonuclease III
CLS10259	DNA mismatch repair ATPase MutS	CLS10130	ribonucleosidediphosphatereductase large subunit
CLS10104	DNA polymerase elongation subunit family B	CLS10252	ribonucleosidediphosphatereductase small subunit
CLS10201	DNA topoisomerase IB	CLS10028	TATA-box-binding protein
CLS10230	DNA topoisomerase II	CLS10057	Transcription factor S-II (TFIIS)-domain-containing protein
CLS10090	DNA-dependent RNA polymerase subunit Rpb9/M	CLS10055	transcription initiation factor IIB
CLS10250	DNA-directed RNA polymerase subunit 5 (RPB5)	CLS10011	ubiquitin-conjugating enzyme E2
CLS10261	DNA-directed RNA polymerase subunit 6	CLS10214	Ulp1-like protease
CLS10076	DNA-directed RNA polymerase subunit alpha	CLS10066	VV A18-like helicase
CLS10053	DNA-directed RNA polymerase subunit beta	CLS10068	VV A32 virion packaging ATPase
CLS10249	DNA-directed RNA polymerase subunit E’ (RPB7)	CLS10218	YqaJ-like viral recombinase
CLS10024	Erv1 / Alr family oxidoreductase	CLS10212	hypothetical protein
CLS10221	eukaryotic translation initiation factor 4E-like protein	CLS10222	hypothetical protein
CLS10086	FtsJ-like methyltransferase	CLS10233	hypothetical protein
CLS10030	Holliday junction resolvase	CLS10236	hypothetical protein
CLS10056	metallopeptidase WLM	CLS10032	hypothetical protein
CLS10219	mRNA capping enzyme	CLS10043	hypothetical protein
CLS10088	NUDIX hydrolase	CLS10046	hypothetical protein
CLS10224	poxvirus poly(A) polymerase catalytic subunit-like protein	CLS10070	hypothetical protein
CLS10253	probable ubiquitin carboxyl-terminal hydrolase	CLS10081	hypothetical protein
**Genes missing in one or two OLPG genomes but present in all the other *Mimiviridae *genomes**			
CLS10059	AAA family ATPase	CLS10009	Lon domain protease
CLS10021	chaperone protein DnaJ	CLS10033	patatin-like phospholipase
CLS10022	chaperone protein DnaJ	CLS10072	Prolyl 4-hydroxylase
CLS10082	heat shock 70 kDa protein	CLS10091	thymidylate synthase
CLS10042	hypothetical protein	CLS10010	XRN 5'-3' exonuclease

**Figure 3:**
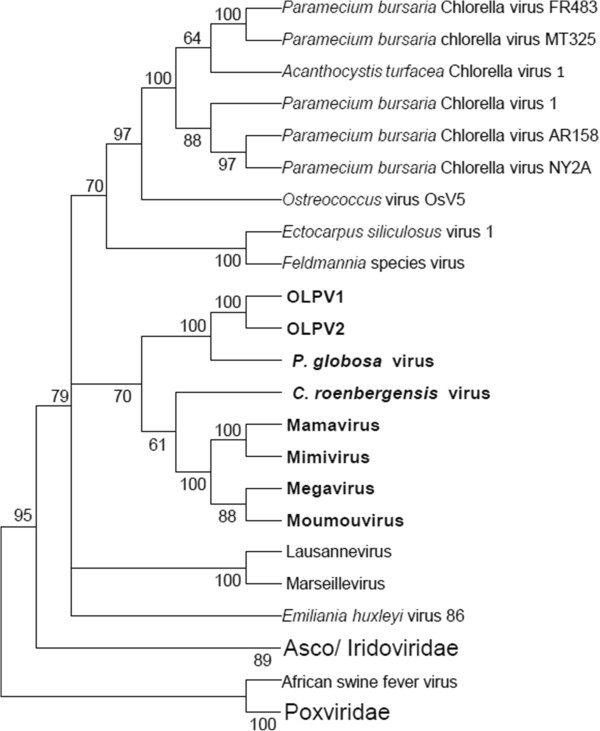
**Neighbor-Joining gene content tree of the NCLDV. **Bootstrap values were obtained by 1,000 resamplings of the initial patterns.

A Neighbor-Joining gene content tree [8,40] was constructed from gene presence-absence patterns in 1,723 mimiCOGs and NCVOGs (Figure 3). In this tree the OLPG forms a clade with the *Mimiviridae* including CroV as the outgroup to the mimiviruses *sensu strictu*. Thus, the similarity of the gene repertoires is compatible with the common ancestry of the OLPG and the Mimiviridae. A maximum likelihood reconstruction of the evolution of the NCLDV [8] assigned nearly 50 viral genes to the ancestral core that presumably dates back to the last common ancestor of all NCLDV although some of the ancestral genes were replaced with xenologs in the course of subsequent evolution [41].

### The Mimiviridae-OLPG clade in the phylogenetic trees of conserved NCLDV genes

We used the mimiCOGs to conduct a new phylogenomic analysis of the ancestral NCLDV genes in an attempt to elucidate the evolutionary affinity of the OLPG (Additional file 3: Table S2). Phylogenetic trees were constructed for all clusters of orthologous genes that included the mimiviruses, OLPG and phycodnaviruses and for which the number of informative sites in the multiple sequence alignment was sufficient for phylogenetic analysis.

### Genes involved in DNA replication, recombination and repair

**Figure 4:**
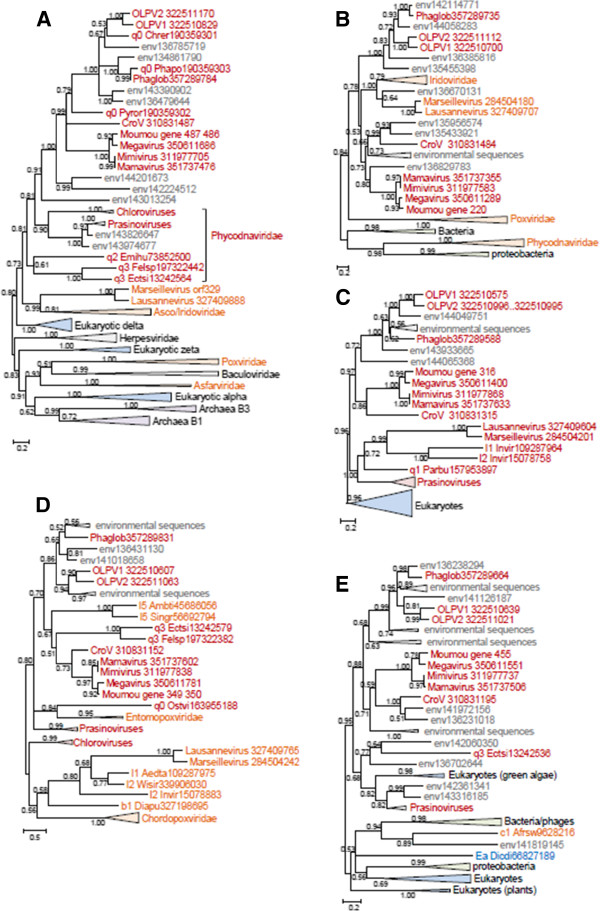
**Maximum-Likelihood trees of ancestral NCLDV genes involved in DNA replication, recombination, and repair. A**, DNA polymerase **B**, D5 primase-helicase. **C**, DNA topoisomerase II. **D**, Holliday junction (RuvC) resolvase. **E**, YqaJ-like recombinase. Branches with bootstrap support less than 0.5 were collapsed. For each sequence, the species name abbreviation and the gene identification numbers are indicated; env stands for “marine metagenome.” Species abbreviations: CroV, *C.roenbergensis *virus; Moumou, Moumouvirus; OLPV1, Organic Lake phycodnavirus 1; OLPV2, Organic Lake phycodnavirus 2; Phaglob, *P. globosa *virus; Aedta, Invertebrate iridescent virus 3; Afrsw, African swine fever virus; Ambti, *Ambystomatigrinum *virus; Chrer, *Chrysochromulina ericina *virus; Diapu, *Diadromuspulchellus *ascovirus 4a; Dicdi, *Dictyostelium discoideum *AX4; l1_Invir, Invertebrate iridescent virus 3; l2_Invir, Invertebrate iridescent virus 6; Ostvi, *Ostreococcus *virus OsV5; Parbu, *Paramecium bursaria *Chlorella virus AR158; Phapo, *Phaeocystis pouchetii *virus; Pyror, *Pyramimonas orientalis *virus; Singr, Singapore grouper iridovirus; Wisir, *Wiseana* iridescent virus; Ectsi, *Ectocarpus siliculosus *virus 1; Emihu, *Emiliania huxleyi* virus 86; Felsp, *Feldmannia sp *virus. Taxa abbreviations: Ea, Amoebozoa; b1, Ascovirus; c1, Asfarviridae; l1, Chloriridovirus; l2, Iridovirus; l5, Ranavirus; q0, unclassified Phycodnaviridae; q1, Chlorovirus; q2, Coccolithovirus; q3, Phaeovirus.

Among 13 genes in this category, 7 are missing in both Phycodnaviruses and the OLPG, suggestive of parallel gene loss (See Additional file 3: Table S2). In the DNA polymerase B tree, the OLPG cluster with mimiviruses with 0.99 bootstrap support (Figure 4A). Three unclassified, partially sequenced viruses, *Chrysochromulina ericina* virus, *Phaeocystis pouchetii* virus, and *Pyramimonas orientalis* virus, also appear to belong to the OLPG group. Phycodnaviruses in this tree are paraphyletic, and topology testing confidently rejects monophyly of OLPG with any one of the two branches of Phycodnaviruses. In contrast, the monophyly of phycodnaviruses is supported, with the respective tree having a slightly greater likelihood than the unconstrained DNAP tree; however, joining OLPG with the single Phycodnavirus branch is rejected as well (Additional file 4: Figure S3).

In the D5 helicase tree (Figure 4B), OLPG and mimiviruses are paraphyletic but form a well-supported clade with iridoviruses and Marseilleviruses whereas phycodnaviruses group with bacteria and bacteriophages, probably as a result of xenologous gene displacement [41].

The phylogenetic tree of DNA topoisomerase II contains a strongly supported OLPG-*Mimiviridae* clade (Figure 4C); the topology of this tree is nearly identical to that of the DNA polymerase tree. The tree of the YqaJ-like recombinase also supports the OLPG-*Mimiviridae* clade (Figure 4D). By contrast, in the tree of RuvC-like Holliday junction resolvases, the OLPG fail to cluster with either phycodnaviruses or mimiviruses (Figure 4E).

### Genes involved in transcription and RNA processing

**Figure 5:**
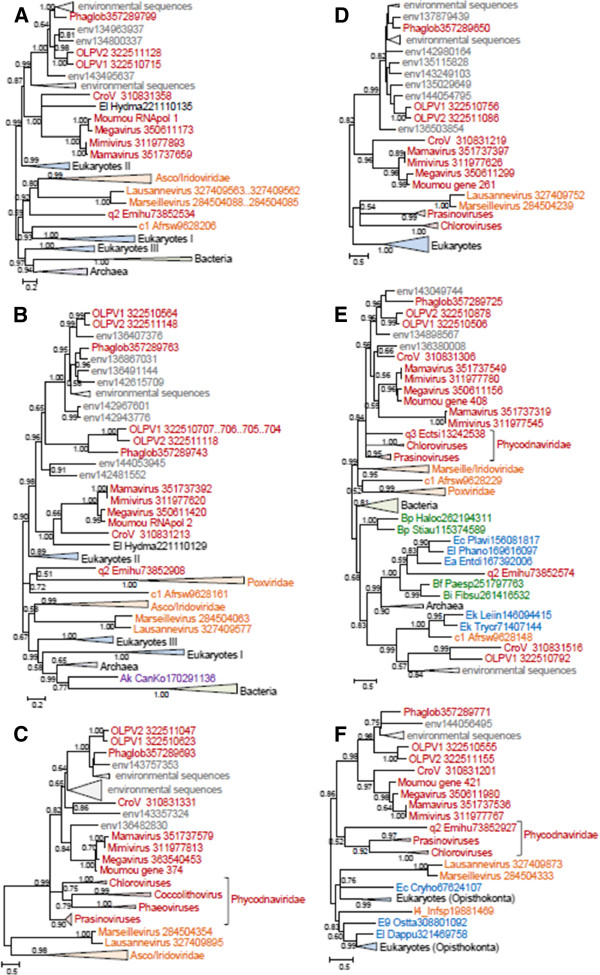
**Maximum-Likelihood trees of ancestral NCLDV genes involved in transcription and RNA processing. A**, RNA polymerase alpha subunit. **B**, RNA polymerase beta subunit. **C**, A2L transcription factor. **D**, Transcription initiation factor TFIIB. **E**, A18-like helicase. **F**, mRNA capping enzyme. Branches with bootstrap support less than 0.5 were collapsed. For each sequence, the species name abbreviation and the gene identification numbers are indicated; env stands for environmental sequences. Species abbreviations:CroV, *C.roenbergensis *virus; Moumou, Moumouvirus; OLPV1, Organic Lake phycodnavirus 1; OLPV2, Organic Lake phycodnavirus 2; Phaglob, *P. globosa *virus; Afrsw, African swine fever virus; CanKo, *Candidatus *Korarchaeum cryptofilum OPF8; Emihu, *Emiliania huxleyi *virus 86; Hydma, *Hydra magnipapillata*. Taxa abbreviations: Ak, Korarchaeota; El, Opisthokonta; c1, Asfarviridae; q2, Coccolithovirus.

The RNA polymerase (RNAP) subunits alpha and beta have been lost in Phycodnaviruses. However, given that the NCLDV are polyphyletic in the phylogenies of both these genes [41], we constructed trees and examined the provenance of the OLPG. In both trees (Figure 5AB), OLPG and the *Mimiviridae* are monophyletic and group with Eukaryotic RNAP II. Notably, the RNAP beta gene is duplicated in OLPG. The phylogenies of other genes encoding proteins involved in transcription and RNA processing including the transcription factors A2_L and TFIIB, A18-like helicase, and capping enzyme (guanylyltransferase domain only because the methyltransferase domain is missing in phycodnaviruses) also showed monophyly of OLPG and the *Mimiviridae* (Figure 5C-F).

**Figure 6:**
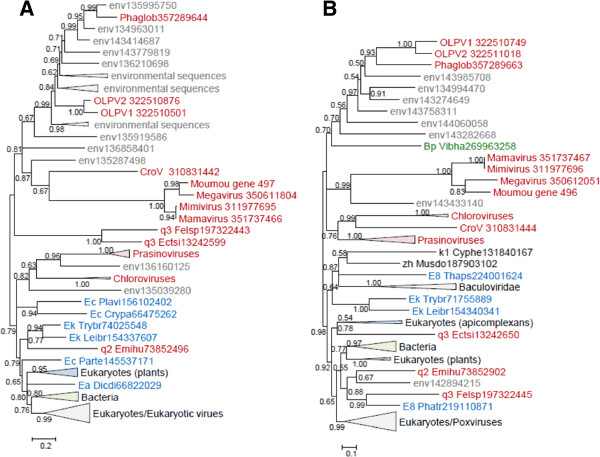
**Maximum-Likelihood trees of ancestral NCLDV genes involved in nucleotide metabolism. A**, Ribonucleoside diphosphate reductase small subunit. **B**, Ribonucleoside diphosphate reductase large subunit. Branches with bootstrap support less than 0.5 were collapsed. For each sequence, the species name abbreviation and the gene identification numbers are indicated; env stands for environmental sequences. Species abbreviations:CroV, C. roenbergensis virus; Moumou, Moumouvirus; OLPV1, Organic Lake phycodnavirus 1; OLPV2, Organic Lake phycodnavirus 2; Phaglob, *P. globosa* virus; Crypa, *Cryptosporidium parvum *Iowa II; Cyphe, Cyprinid herpesvirus 3; Dicdi, *Dictyostelium discoideum *AX4; Ectsi, *Ectocarpus siliculosus *1; Emihu, *Emiliania huxleyi *virus 86; Felsp, *Feldmannia sp *virus; Leibr, *Leishmania braziliensis*; Musdo, *Musca domestica *salivary gland hypertrophy virus; Parte, *Paramecium tetraurelia *strain d4-2; Phatr, *Phaeodactylum tricornutum *CCAP 1055/1; Plavi, *Plasmodium vivax *Sal-1; Thaps, *Thalassiosira pseudonana *CCMP1335; Trybr, *Trypanosoma brucei*; Vibha, *Vibrio harveyi *1DA3. Taxa abbreviations: Bp, Proteobacteria; E8, Stramenopiles; Ea, Amoebozoa; Ec, Alveolata; Ek, Kinetoplastida; k1, Herpesvirales; q2, Coccolithovirus; q3, Phaeovirus; zh, unclassified dsDNA viruses.

Among the genes encoding enzymes of nucleotide metabolism, only those for the two subunits of ribonucleotide reductase were amenable to phylogenetic analysis. The tree for the small subunit supports monophyly of OLPG-*Mimiviridae* (Figure 6A) whereas in the tree for the large subunit the OLPG, the mimivirus and phycodnavirus branches are unresolved (Figure 6B).

**Figure 7:**
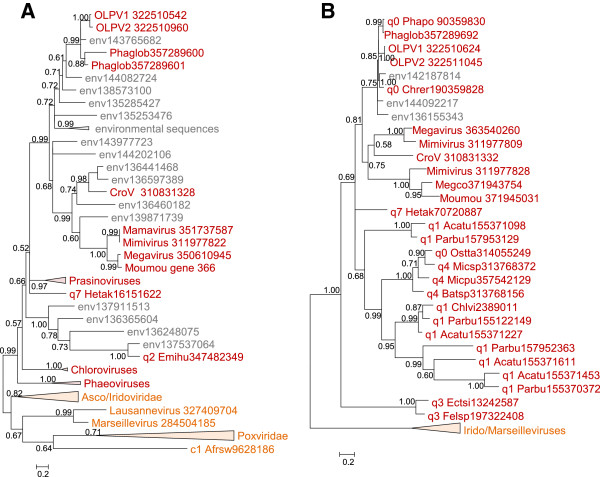
**Maximum-Likelihood trees of two genes involved in virion structure and morphogenesis. A**, A32 virion packaging ATPase. **B**, Major capsid protein. Branches with bootstrap support less than 0.5 were collapsed. For each sequence, the species name abbreviation and the gene identification numbers are indicated; env stands for “marine metagenome.” Species abbreviations: Acatu, *Acanthocystis turfacea *Chlorella virus 1; Afrsw, African swine fever virus; Batsp, *Bathycoccus *sp. RCC1105 virus BpV1; Chlvi, Chlorella virus; Chrer, *Chrysochromulina ericina *virus; CroV , *C. roenbergensis *virus; Ectsi, *Ectocarpus siliculosus *virus 1; Emihu, *Emiliania huxleyi *virus 86; Felsp, *Feldmannia *species virus; Hetak, *Heterosigma akashiwo *virus 01; Megco, Megavirus courdo7; Micpu, *Micromonas pusilla *virus SP1; Micsp, *Micromonas *sp. RCC1109 virus MpV1; Moumou, Moumouvirus; OLPV1, Organic Lake phycodnavirus 1; OLPV2, Organic Lake phycodnavirus 2; Ostta, *Ostreococcus tauri* virus 2; Parbu, *Paramecium bursaria *Chlorella virus AR158; Phaglob, *P. globosa *virus; Phapo, *Phaeocystis pouchetii *virus. Taxa abbreviations: c1, Asfarviridae; q0, unclassified Phycodnaviridae; q1, Chlorovirus; q2, Coccolithovirus; q3, Phaeovirus; q4, Prasinovirus; q7, Raphidovirus.

The only phylogenetic tree that was obtained for a gene encoding a protein involved in virion morphogenesis, the A32-like DNA packaging ATPase, also supports the OLPG-*Mimiviridae* monophyly (Figure 7A).

The phylogenetic analysis of the Major Coat Protein (MCP) gene required a modified approach because the mimiviruses [18,19] as well as OLPG [33] encompass multiple paralogous MCP genes some of which are extremely diverged in sequence [18,19], hampering the construction of robust phylogenetic trees. Therefore we first aligned all detected MCP sequences from *Mimiviridae*, OLPG, *Phycodnaviridae*, *Iridoviridae* and *Marseilleviridae* (the sequences from *Asfarviridae* and *Poxviridae* being in this case too distant) and constructed a preliminary phylogenetic tree. This tree was used to identify the fastest evolving MCP homologs (the longest branches) which were then removed from the sequence alignment that was when used to construct the final phylogenetic tree. In this MCP phylogeny, the OLPG-Mimiviridae clade was recovered with moderate statistical support (Figure 7B).

**Figure 8:**
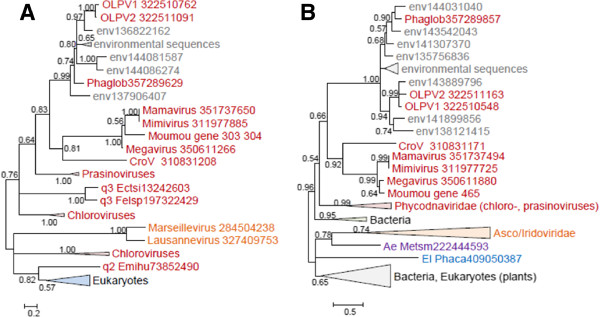
**Maximum-Likelihood phylogenetic trees of two genes ancestral to the Mimi-,Asco/Irido-Phycodna-, and Marseilleviruses. A**, Proliferating cell nuclear antigen. **B**, ribonuclease III. Branches with bootstrap support less than 0.5 were collapsed. For each sequence, the species name abbreviation and the gene identification numbers are indicated; env stands for “marine metagenome.” Species abbreviations:CroV, *C. roenbergensis *virus; Moumou, Moumouvirus; OLPV1, Organic Lake phycodnavirus 1; OLPV2, Organic Lake phycodnavirus 2; Phaglob, *P. globosa *virus; Ectsi, *Ectocarpus siliculosus *virus 1; Emihu, *Emiliania huxleyi *virus 86; Felsp, *Feldmannia *species virus; Metsm , *Methanobrevibacter smithii *DSM 2375; Phaca, *Phanerochaete carnosa. *Taxa abbreviations: Ae, Euryarchaeota; El, Opisthokonta; q2, Coccolithovirus; q3, Phaeovirus.

In addition, we examined the set of genes that are projected to the last common ancestor of the major branch of the NCLDV that consists of *Iridoviridae*, *Marseilleviridae*, *Mimiviridae* and *Phycodnaviridae*[8] and obtained phylogenetic trees for two of these genes, those for the Proliferating Cell Nuclear Antigen (PCNA)-like replication factor and ribonuclease III. Both trees support the OLPG-*Mimiviridae* monophyly (Figure 8AB).

### Reconstruction of the evolution of giant viruses

**Figure 9:**
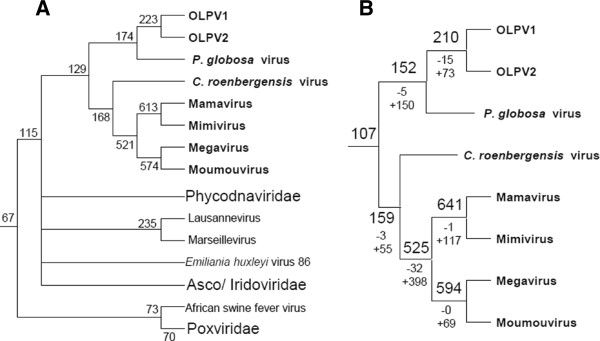
**Maximum-Likelihood reconstruction of gene loss and gene gain events in the evolution of the NCLDV. **The tree from Figure 3 was used as a guide for the reconstruction. **A**. The inferred numbers of genes present in each node are shown. **B**. Numbers of mimiCOGs present with the likelihood greater than 0.9. Numbers after plus and minus signs represent numbers of genes gained or lost since the previous node.

The phyletic patterns of the amended NCVOGs were superimposed on the Neighbor-Joining gene content tree (Figure 3) and employed to produce a new maximum likelihood reconstruction of gene gain and loss in the NCLDV [8]. The reconstruction of the ancestral gene repertoires based solely on phyletic patterns is to be viewed with caution given the complexity of the evolution of the NCLDV that on some occasions apparently involved parallel gains of homologous genes [41] as well as the inherent probabilistic nature of the reconstruction [8,42]. Nevertheless, the results clearly indicate some limited gene gain in the OLPG contrasted by massive gene gain in both the *Mimiviridae* branch and the mimiviruses *sensu strictu*, after their radiation from the common ancestor with the CroV (Figure 9AB; Additional file 4: Figure S4). This extensive gene gain in the mimiviruses, along with the considerable diversity of the gene repertoires even among closely related mimiviruses (Mimivirus, Moumouvirus and Megavirus [30] implies a large, “open” pangenome of these giant viruses [43]. Conceivably, this expansive pangenome evolved through numerous acquisitions and exchanges of genes between diverse members of the vast intracellular microbiomes of phagotrophic amoeba that include bacteria, fungi and viruses [30,44,45]. Apparently, the extensive horizontal gene transfer within this microbiome results in mosaic gene repertoires of amoebal viruses as observed both in mimiviruses [14] and Marseillevirus [46]. Gene transfers are likely to be facilitated by the mobilome of the giant viruses that includes virophages as well as transpovirons, a distinct group of linear plasmids [30]. The OLPV, CroV and possibly other members of the extended family *Mimiviridae* that reproduce in hosts colonized by fewer microbes appear to possess smaller (pan)genomes and lower degrees of genomic mosaicism [22,33]. Thus, the size and diversity of the pangenomes of large viruses seem to strongly depend on the life styles of their hosts.

## Conclusions

Taken together, the phylogenomic results presented here indicate that the OLPG are the sister group of the family *Mimiviridae* within the NCLDV phylogeny. This conclusion is supported by the topologies of the phylogenetic trees for most of the core NCLDV genes that show monophyly of OLPG and the mimiviruses (Figures 4, 5, 6, 7, 8 and Additional file 3: Table S2). Although some of the phylogenies are poorly resolved, none of them shows clustering of the OLPG with or within the phycodnaviruses. Moreover, for some of the core NCLDV genes, conservative statistical tests reject affiliation of OLPG with Phycodnaviruses. Given that the OLPG, at least so far, are a group with limited diversity, it seems plausible that eventually the family *Mimiviridae* is expanded to include these viruses. Alternatively, OLPG could become a new family within the proposed order *Megavirales*[9].

The OLPG encompass few genes encoding translation system components that are one of the signatures of the mimivirus genomes [14,21] (the only translation-related gene that was apparently acquired by the common ancestor of the OLPG and the mimiviruses is the homolog of the elongation factor 2E) indicating that these genes largely were acquired by an ancestral mimivirus.

An Organic Lake “phycodnavirus” has been identified as a host to a distinct virophage (OLV) [33] that is distantly related to the Sputnik virophage infecting mimiviruses [16,31] and the Mavirus virophage infecting CroV [32]. The findings described here indicate that so far only viruses within the (extended) family *Mimiviridae* support the reproduction of virophages. Recently, numerous sequences of putative virophages have been assembled from metagenomics sequences originating from diverse environments [47]. In particular, 4 complete virophage genomes distantly related to the OLV have been assembled from Yellowstone Lake metagenomic data. The presents results lead us to hypothesize that these novel virophages also infect member of the family Mimiviridae, in particular still unknown representatives of the OLPG group.

Finally, it is worth noting that the mimiCOGs developed in the course of this work are expected to become a key resource for a comprehensive phylogenomic study of the giant viruses, and in particular a full assessment of the fourth domain hypothesis.

## Methods

### MimiCOG construction

For the construction of mimiCOGs, the following genomes were downloaded from GenBank (http://www.ncbi.nlm.nih.gov/): *Acanthamoeba polyphaga* mimivirus (GI:311977355), *Acanthamoeba castellanii* mamavirus (GI:351737110), *Megavirus chiliensis* (GI:350610932), *Cafeteria roenbergensis* virus BV-PW1 (GI:310830989), *Phaeocystis globosa* virus 12 T (GI: 357289534), Organic Lake phycodnavirus 1 (GI:322510471),Organic Lake phycodnavirus 2 (GI:322510873), Marseillevirus (GI:284504040), and Lausannevirus (GI:327409548). The complete dataset consisted of 6,548 protein sequences. The mimiCOGs were constructed as previously described [38]. Briefly, the procedure included the following steps: 1) Initial clusters based on triangles of symmetrical best hits were constructed using a modified COG algorithm using as the input the results of all-against-all BLASTP [48] comparison; 2) Multiple alignments of the initial cluster members were constructed using the MUSCLE program [49]. The alignments were used to generate position-specific scoring matrices (PSSM) for a PSI-BLAST search [48] against the original protein dataset. Significantly similar proteins were added to the corresponding clusters; 3) Clusters with nearly complementary phyletic patterns and high inter-cluster sequence similarity were manually examined and merged whenever appropriate; 4) The mimiCOGs were manually edited and annotated using annotations of Moumouvirus and Mamavirus proteins present and RPS-BLAST [50] and PSI-BLAST of other cluster members; 5) MimiCOG-NCVOG correspondence was established by PSI-BLAST search initiated with PSSMs constructed from NCVOG alignments [8] against proteins included in the mimiCOGs. The mimiCOGs are available at ftp://ftp.ncbi.nih.gov/pub/koonin/mimivirus/mimiCOGs.

### Neighbor-Joining tree based on the phyletic patterns

Presence-absence matrices of mimiCOGs and corresponding NCVOGs were combined, whenever correspondence was established, and binarized yielding 584 patterns (see Additional file 5). Nineteen NCVOG patterns were amended by adding OLPG proteins that have not been included in the mimiCOGs based on the result of PSI-BLAST searches initiated by NCVOG PSSMs against proteins used for mimiCOG construction. The remaining 727 NCVOGs and 393 mimiCOGs were considered non-overlapping and added to the pool resulting in the total of 1,723 patterns. For each pair of species the number of clusters where each of them were present (*N*1 and *N*2) as well as the number of clusters where both species were present (*N*U) were computed. The gene content similarity measure (*s*) was calculated as *s* = *N*U/sqrt(*N*1 × *N*2) and converted to a distance measure (*d*) as *d* = -ln(*s*)[8]. A neighbor-joining tree was constructed from the distance matrices using the NEIGHBOR program of Phylip 3.66 [51]. Bootstrap values were obtained by 1,000 resamplings of the 1,723 patterns.

### Multiple alignment and phylogenetic tree construction

The sequences for phylogenetic analysis were collected using (i) BLAST searches against nr and environmental (env_nr) databases initiated by distant mimiCOG members; (ii) the corresponding NCVOG sequences [8]; and (iii) reference sequences used for the core NCVOG study [41]. Nearly identical sequences were eliminated using BLASTCLUST (http://www.ncbi.nlm.nih.gov/IEB/ToolBox/C_DOC/lxr/source/doc/blast/blastclust.html). The sequences were aligned using MUSCLE [49]. All alignments were manually checked for the conservation of domain architecture and presence of diagnostic motifs. Positions including gaps in more than one-third of the sequences and positions with low information content were removed prior to tree computation [52]. A preliminary maximum-likelihood tree was constructed using the FastTree program with default parameters (JTT evolutionary model, discrete gamma model with 20 rate categories; [53]). The preliminary tree and the alignment were then used to determine the best substitution matrix using Prottest [54]. Final maximum-likelihood trees were constructed using TreeFinder (1,000 replicates, Search Depth 2 [55]), with the substitution matrix found to be the best for a given alignment. The Expected-Likelihood Weights (ELW) of 1,000 local rearrangements were used as confidence values of TreeFinder tree branches. For topology testing, whenever applicable, alternative (constrained) topologies were constructed and compared to the initial trees using TreeFinder. Approximately unbiased (AU) test P value cutoff 0.05 was used for rejecting tree topologies [56].

### Reconstruction of gene losses and gains

The Neighbor-Joining gene content tree of the NCLDV and the gene presence-absence matrix for the mimiCOGs and NCVOGs were used to reconstruct the gene loss and gain events in the evolution of the NCLDV using the COUNT program [42], as previously described [8].

## Competing interests

The authors declare that they have no competing interests.

## Authors’ contributions

NY collected the data; NY, PC, DR and EVK analyzed the data; NY and EVK wrote the manuscript that was read and approved by all authors.

## Supplementary Material

Additional file 1 Annotation of the OLPG genes. Click here for file

Additional file 2 The mimiCOGs. Click here for file

Additional file 3: Table S2 Phyletic patterns and major inferred evolutionary events for ancestral NCVOGs. Click here for file

Additional file 4 Topology testing results for selected phylogenetic trees of NCLDV genes. Click here for file

Additional file 5: Phyletic patterns used for the reconstruction of gene gain and gene loss in the NCLDV. Click here for file
