# Supplementary material for: Mimiviridae: clusters of orthologous genes, reconstruction of gene repertoire evolution and proposed expansion of the giant virus family
Source: Virol J. 2013 Apr 4;10:106. doi: 10.1186/1743-422X-10-106 (PMC3620924; doi:10.1186/1743-422X-10-106)
Supplement: Additional file 3: Table S2 — Phyletic patterns and major inferred evolutionary events for ancestral NCVOGs. [file 1743-422X-10-106-S3.doc]

|  |  |  | Asco/Iridoviruses (out of 11) | marseillevirus (1) | mimiviruses (out of 2) | phycodnaviruses (out of 10) | poxviruses (out of 20) | asfarviruses (1) | Acanthamoeba_castellanii_mamavirus | Acanthamoeba_polyphaga_mimivirus | Megavirus_chiliensis | Moumouvirus | Cafeteria_roenbergensis_virus | Organic_Lake_phycodnavirus_1 | Organic_Lake_phycodnavirus_2 | Phaeocystis_globosa_virus_12T | Lausannevirus | Marseillevirus |  |
| --- | --- | --- | --- | --- | --- | --- | --- | --- | --- | --- | --- | --- | --- | --- | --- | --- | --- | --- | --- |
| *NCVOG* | *mimiCOG* | *mimiCOG annotation* | *# of genomes in NCVOG* | | | | | | *# of genomes in mimiCOG* | | | | | | | | | | *comments* |
| **DNA replication, recombination and repair** | | | | | | | | | | | | | | | | | | | |
| NCVOG0038 | CLS10104 | DNA polymerase family B | 11 | 1 | 2 | 10 | 20 | 1 | 1 | 1 | 1 | 1 | 1 | 1 | 1 | 1 | 1 | 1 | monophyletic OLPG-CroV-mimiviruses |
| NCVOG0023 | CLS10262 | D5-like helicase-primase | 11 | 1 | 2 | 10 | 20 | 1 | 1 | 1 | 1 | 1 | 1 | 1 | 1 | 1 | 1 | 1 | non-orthologous gene displacement in Phycodnaviridae |
| NCVOG1060 | CLS10193 | FLAP-like endonuclease XPG | 11 | 1 | 2 | 1 | 20 |  | 1 | 1 | 1 | 1 | 1 |  |  |  | 1 | 1 | loss in both OLPG and phycodnaviruses |
| NCVOG0036 | CLS10027 | DNA topoisomerase 1b |  |  | 2 | 1 | 20 |  | 1 | 1 | 1 | 1 | 1 |  |  |  |  |  | loss in both OLPG and phycodnaviruses |
| NCVOG0037 | CLS10230 | DNA topoisomerase II | 2 | 1 | 2 | 8 | 1 | 1 | 1 | 1 | 1 | 1 | 1 | 1 | 1 | 1 | 1 | 1 | monophyletic OLPG-CroV-mimiviruses |
| NCVOG0035 | CLS10255 | NAD-dependent DNA ligase | 2 |  | 2 |  | 3 |  | 1 | 1 | 1 | 1 | 1 |  |  | 1 |  |  | loss in phycodnaviruses |
| NCVOG0034 | CLS10270 | ATP-dependent DNA ligase |  | 1 |  | 6 | 11 | 1 |  |  |  |  |  | 1 |  |  | 1 | 1 | loss in CroV and mimiviruses |
| NCVOG0278 | CLS10030 | Holliday junction resolvase | 4 | 1 | 2 | 9 | 20 |  | 1 | 1 | 1 | 1 | 1 | 1 | 1 | 1 | 1 | 1 | tree: OLPGis not monophyletic neither with phycodna- nor with mimiviruses |
| NCVOG0004 | CLS10273 | AP (apurinic) endonuclease |  | 1 | 2 |  | 2 | 1 | 1 | 1 | 1 | 1 |  |  |  |  | 1 | 1 | independent losses in OLPG and phycodnaviruses |
| NCVOG0004a | CLS10254 | DNA polymerase family X |  |  |  |  |  |  | 1 | 1 | 1 | 1 | 1 |  |  | 1 |  |  | independent losses in OLPG and phycodnaviruses |
| NCVOG1192 | CLS10218 | YqaJ-like viral recombinase |  |  | 2 | 9 | 1 | 1 | 1 | 1 | 1 | 1 | 1 | 1 | 1 | 1 |  |  | monophyletic OLPG-CroV-mimiviruses |
| NCVOG0024 | CLS10277 | replication origin-binding helicase |  | 1 | 2 |  |  | 1 | 1 | 1 |  | 1 |  |  |  |  |  |  | Independent losses in OLPG and phycodnaviruses |
| NCVOG1115 | CLS10275 | uracil-DNA glycosylase |  | 1 | 2 |  | 20 |  | 1 | 1 | 1 | 1 |  |  |  |  | 1 | 1 | Independent losses in OLPG and phycodnaviruses |
| **Transcription and RNA processing** | | | | | | | | | | | | | | | | | | | |
| NCVOG0274 | CLS10076 | DNA-directed RNA polymerase subunit alpha | 11 | 1 | 2 | 1 | 20 | 1 | 1 | 1 | 1 | 1 | 1 | 1 | 1 | 1 | 1 | 1 | loss in Phycodnaviruses; OLPG and mimiviruses cluster with eukaryotic RNA polymerase II |
| NCVOG0271 | CLS10053 | DNA-directed RNA polymerase subunit beta | 11 | 1 | 2 | 1 | 20 | 1 | 1 | 1 | 1 | 1 | 1 | 1 | 1 | 1 | 1 | 1 | loss in Phycodnaviruses; OLPG and mimiviruses cluster with eukaryotic RNA polymerase II |
| NCVOG0273 | CLS10250 | DNA-directed RNA polymerase subunit 5 (RPB5) | 10 | 1 | 2 | 1 |  | 1 | 1 | 1 | 1 | 1 | 1 | 1 | 1 | 1 | 1 | 1 | loss in Phycodnaviruses; insufficient sequence conservation for reliable phylogenetic analysis |
| NCVOG1164 | CLS10031 | A1L transcription factor VLTF-2 | 10 | 1 | 2 | 10 | 20 | 1 | 1 | 1 | 1 | 1 | 1 | 1 | 1 | 1 | 1 | 1 | not enough sequence conservation for reliable phylogenetic analysis |
| NCVOG0262 | CLS10071 | A2L transcription factor VLTF-3 | 11 | 1 | 2 | 10 | 20 | 1 | 1 | 1 | 1 | 1 | 1 | 1 | 1 | 1 | 1 | 1 | monophyletic OLPG-CroV-mimiviruses |
| NCVOG0261 | CLS10225 | poxvirus early transcription factor-like protein | 11 | 1 | 2 |  | 20 | 1 | 1 | 1 | 1 | 1 | 1 |  |  |  | 1 | 1 | Independent losses in OLPG and phycodnaviruses |
| NCVOG0272 | CLS10057 | Transcription factor S-II (TFIIS)-domain-containing protein | 9 | 1 | 2 | 8 | 18 | 1 | 1 | 1 | 1 | 1 | 1 | 1 | 1 | 1 | 1 | 1 | insufficient sequence conservation for reliable phylogenetic analysis |
| NCVOG1127 | CLS10055 | transcription initiation factor TFIIB |  | 1 | 2 | 7 |  | 1 | 1 | 1 | 1 | 1 | 1 | 1 | 1 | 1 | 1 | 1 | monophyletic OLPG-CroV-mimiviruses |
| NCVOG0076 | CLS10066 | VV A18-like helicase | 5 | 1 | 2 | 9 | 20 | 1 | 1 | 1 | 1 | 1 | 1 | 1 | 1 | 1 | 1 | 1 | monophyletic OLPG-CroV-mimiviruses |
| NCVOG0267 | CLS10205 | RNA-helicase DExH-NPH-II |  |  | 2 |  | 20 | 1 | 1 | 1 | 1 | 1 | 1 |  |  |  |  |  | Independent losses in OLPG and phycodnaviruses |
| NCVOG1117 | CLS10219 | mRNA capping enzyme | 1 | 1 | 2 | 8 | 20 | 1 | 1 | 1 | 1 | 1 | 1 | 1 | 1 | 1 | 1 | 1 | monophyletic OLPG-CroV-mimiviruses |
| NCVOG0236 | CLS10088 | NUDIX hydrolase | 4 | 1 | 2 | 1 | 20 | 1 | 1 | 1 | 1 | 1 | 1 | 1 | 1 | 1 | 1 | 1 | loss in phycodnaviruses |
| NCVOG1088 | CLS10173 | RNA ligase | 11 | 1 |  |  |  | 1 |  |  |  |  | 1 |  |  | 1 | 1 | 1 | loss in phycodnaviruses |
| - | CLS10827 | Poly A polymerase regulatory subunit |  |  |  |  |  |  | 1 |  | 1 |  |  |  |  |  |  |  | loss in phycodna- and mimiviruses |
| **Nucleotide metabolism** | | | | | | | | | | | | | | | | | | | |
| NCVOG0276 | CLS10252 | ribonucleosidediphosphatereductase small subunit | 2 | 1 | 2 | 10 | 13 | 1 | 1 | 1 | 1 | 1 | 1 | 1 | 1 | 1 | 1 | 1 | monophyletic OLPG-CroV-mimiviruses |
| NCVOG1353 | CLS10130 | ribonucleosidediphosphatereductase large subunit | 7 | 1 | 2 | 10 | 3 | 1 | 1 | 1 | 1 | 1 | 1 | 1 | 1 | 1 | 1 | 1 | OLPG fails to cluster with either mimiviruses or phycodnaviruses |
| NCVOG0319 | CLS10211 | Thymidine kinase |  | 1 | 2 | 1 | 15 | 1 | 1 | 1 | 1 | 1 | 1 |  |  | 1 | 1 | 1 | loss in phycodnaviruses |
| NCVOG0320 | CLS11087 | deoxynucleoside kinase /Thymidylate kinase | 11 |  |  | 5 | 4 | 1 |  |  |  |  |  |  | 1 | 1 |  |  | loss in CroV and mimiviruses |
| NCVOG1068 | CLS10013 | dUTPase | 4 |  |  | 8 | 17 | 1 |  |  |  |  | 1 | 1 | 1 | 1 | 1 |  | loss in mimiviruses |
| **Virion structure and morphogenesis** | | | | | | | | | | | | | | | | | | | |
| NCVOG0022 | CLS10039 | capsid protein | 11 | 1 | 2 | 10 | 20 | 1 | 1 | 1 | 1 | 1 | 1 | 1 | 1 | 1 | 1 | 1 | numerous paralogs; when the most conserved family members are analyzed, the OLPG-CroV-mimiviruses clade is recovered |
| NCVOG0249 | CLS10068 | VV A32 virion packaging ATPase | 11 | 1 | 2 | 10 | 20 | 1 | 1 | 1 | 1 | 1 | 1 | 1 | 1 | 1 | 1 | 1 | monophyletic OLPG-CroV-mimiviruses |
| NCVOG0211 | CLS10383 | Myristylated IMV envelope protein | 11 |  | 2 |  | 20 | 1 | 1 | 1 | 1 | 1 |  |  |  |  |  |  | loss in both OLPGandphycodnaviruses |
| NCVOG1122 | CLS10302 | virion-associated membrane protein | 9 |  | 2 |  | 20 |  | 1 | 1 | 1 | 1 |  |  |  |  |  |  | loss in both OLPGandphycodnaviruses |
| NCVOG0052 | CLS10024 | Erv1 / Alr family oxidoreductase | 11 | 1 | 2 | 9 | 20 | 1 | 1 | 1 | 1 | 1 | 1 | 1 | 1 | 1 | 1 | 1 | numerous paralogs |
| NCVOG0256 | CLS10153 | Glycosyltransferase |  |  | 2 |  | 20 |  | 1 | 1 | 1 | 1 |  | 1 |  | 1 |  |  | loss in CroV, OLPV2, phycodnaviruses |
| NCVOG0246 | CLS10214 | Ulp1-like protease |  |  | 2 | 1 |  | 1 | 1 | 1 | 1 | 1 | 1 | 1 | 1 | 1 |  |  | loss in phycodnaviruses |
| **Signal transduction, regulation** | | | | | | | | | | | | | | | | | | | |
| NCVOG0040 | CLS10516 | tyrosine-protein phosphatase | 3 |  | 2 | 6 | 19 |  | 1 | 1 | 1 | 1 |  |  |  |  | 1 | 1 | loss in OLPG |
| NCVOG0330 | CLS10197 | RING-finger-containing E3 ubiquitin ligase | 3 | 1 | 2 | 4 | 16 |  | 1 | 1 | 1 | 1 | 1 |  |  |  | 1 | 1 | loss in phycodnaviruses |
| NCVOG0329 | CLS10011 | ubiquitin-conjugating enzyme E2 |  |  | 2 |  |  | 1 | 1 | 1 | 1 | 1 | 1 | 1 | 1 | 1 |  |  | loss in phycodnaviruses |
| **Uncharacterized** | | | | | | | | | | | | | | | | | | | |
| NCVOG0059 | CLS10086 | FtsJ-like methyltransferase |  |  | 2 |  |  | 1 | 1 | 1 | 1 | 1 | 1 | 1 | 1 | 1 |  |  | loss in phycodnaviruses |
