# Supplementary material for: Mimiviridae: clusters of orthologous genes, reconstruction of gene repertoire evolution and proposed expansion of the giant virus family
Source: Virol J. 2013 Apr 4;10:106. doi: 10.1186/1743-422X-10-106 (PMC3620924; doi:10.1186/1743-422X-10-106)
Supplement: Additional file 4 — Topology testing results for selected phylogenetic trees of NCLDV genes. [file 1743-422X-10-106-S4.pptx]

## Slide 1
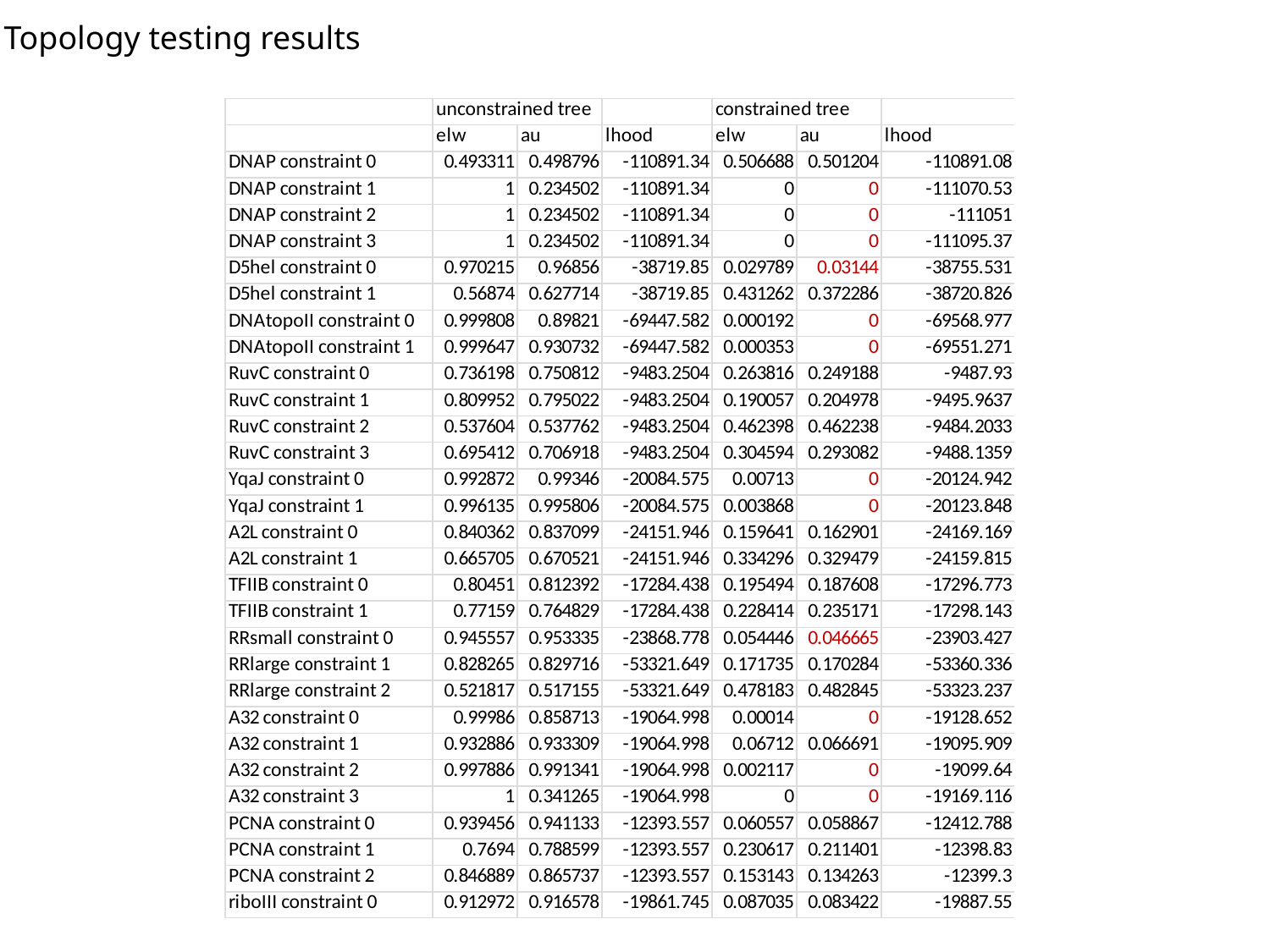

Topology testing results

## Slide 2
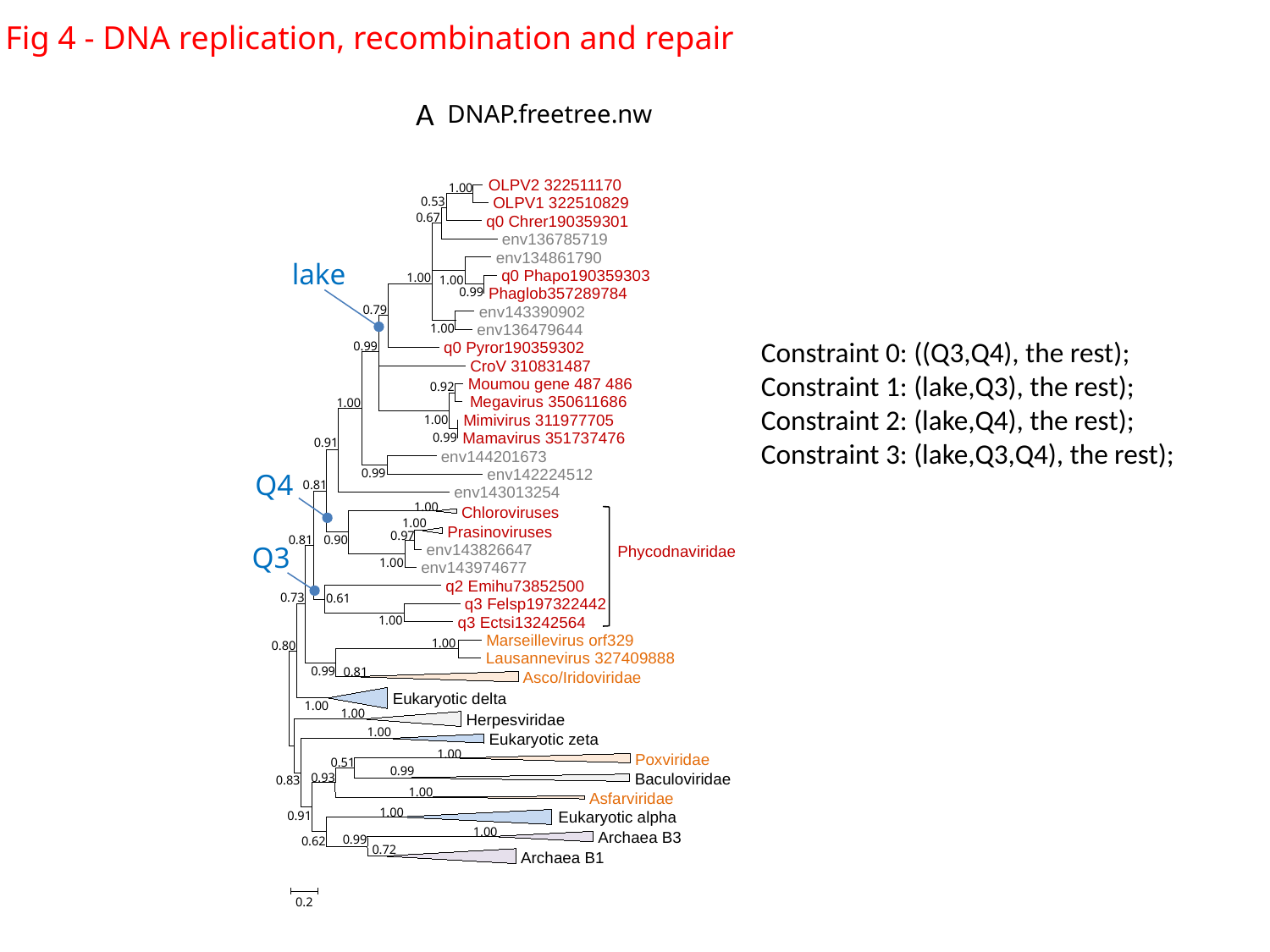

Fig 4 - DNA replication, recombination and repair
A
 DNAP.freetree.nw
 OLPV2 322511170
1.00
 OLPV1 322510829
0.53
0.67
 q0 Chrer190359301
 env136785719
 env134861790
 q0 Phapo190359303
1.00
1.00
 Phaglob357289784
0.99
 env143390902
0.79
 env136479644
1.00
 q0 Pyror190359302
0.99
 CroV 310831487
 Moumou gene 487 486
0.92
 Megavirus 350611686
1.00
 Mimivirus 311977705
1.00
 Mamavirus 351737476
0.99
0.91
 env144201673
 env142224512
0.99
0.81
 env143013254
1.00
 Chloroviruses
1.00
 Prasinoviruses
0.97
0.81
0.90
 env143826647
 Phycodnaviridae
1.00
 env143974677
 q2 Emihu73852500
0.73
0.61
 q3 Felsp197322442
 q3 Ectsi13242564
1.00
 Marseillevirus orf329
1.00
0.80
 Lausannevirus 327409888
0.99
0.81
 Asco/Iridoviridae
 Eukaryotic delta
1.00
1.00
 Herpesviridae
1.00
 Eukaryotic zeta
1.00
 Poxviridae
0.51
0.99
 Baculoviridae
0.93
0.83
1.00
 Asfarviridae
1.00
 Eukaryotic alpha
0.91
1.00
 Archaea B3
0.99
0.62
0.72
 Archaea B1
0.2
lake
Constraint 0: ((Q3,Q4), the rest);
Constraint 1: (lake,Q3), the rest);
Constraint 2: (lake,Q4), the rest);
Constraint 3: (lake,Q3,Q4), the rest);
Q4
Q3

## Slide 3
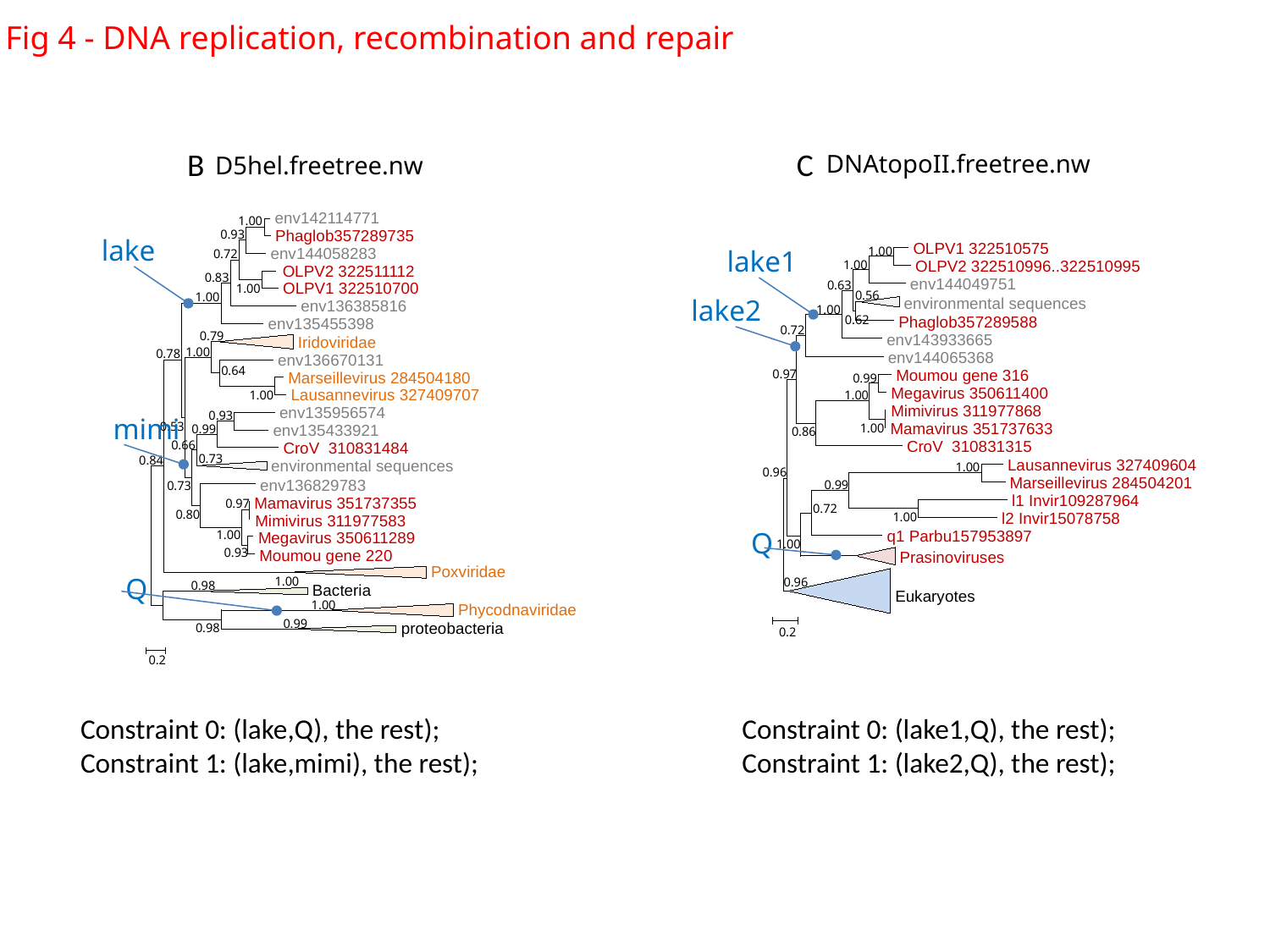

Fig 4 - DNA replication, recombination and repair
B
 D5hel.freetree.nw
C
 DNAtopoII.freetree.nw
 env142114771
1.00
 Phaglob357289735
0.93
 env144058283
0.72
 OLPV2 322511112
0.83
 OLPV1 322510700
1.00
1.00
 env136385816
 env135455398
0.79
 Iridoviridae
1.00
0.78
 env136670131
0.64
 Marseillevirus 284504180
 Lausannevirus 327409707
1.00
 env135956574
0.93
0.53
 env135433921
0.99
0.66
 CroV 310831484
0.73
0.84
 environmental sequences
 env136829783
0.73
 Mamavirus 351737355
0.97
0.80
 Mimivirus 311977583
1.00
 Megavirus 350611289
0.93
 Moumou gene 220
 Poxviridae
1.00
0.98
 Bacteria
1.00
 Phycodnaviridae
0.99
 proteobacteria
0.98
0.2
lake
 OLPV1 322510575
1.00
 OLPV2 322510996..322510995
1.00
 env144049751
0.63
0.56
 environmental sequences
1.00
 Phaglob357289588
0.62
0.72
 env143933665
 env144065368
 Moumou gene 316
0.97
0.99
 Megavirus 350611400
1.00
 Mimivirus 311977868
 Mamavirus 351737633
1.00
0.86
 CroV 310831315
 Lausannevirus 327409604
1.00
0.96
 Marseillevirus 284504201
0.99
 l1 Invir109287964
0.72
 l2 Invir15078758
1.00
 q1 Parbu157953897
1.00
 Prasinoviruses
0.96
 Eukaryotes
0.2
lake1
lake2
mimi
Q
Q
Constraint 0: (lake,Q), the rest);
Constraint 1: (lake,mimi), the rest);
Constraint 0: (lake1,Q), the rest);
Constraint 1: (lake2,Q), the rest);

## Slide 4
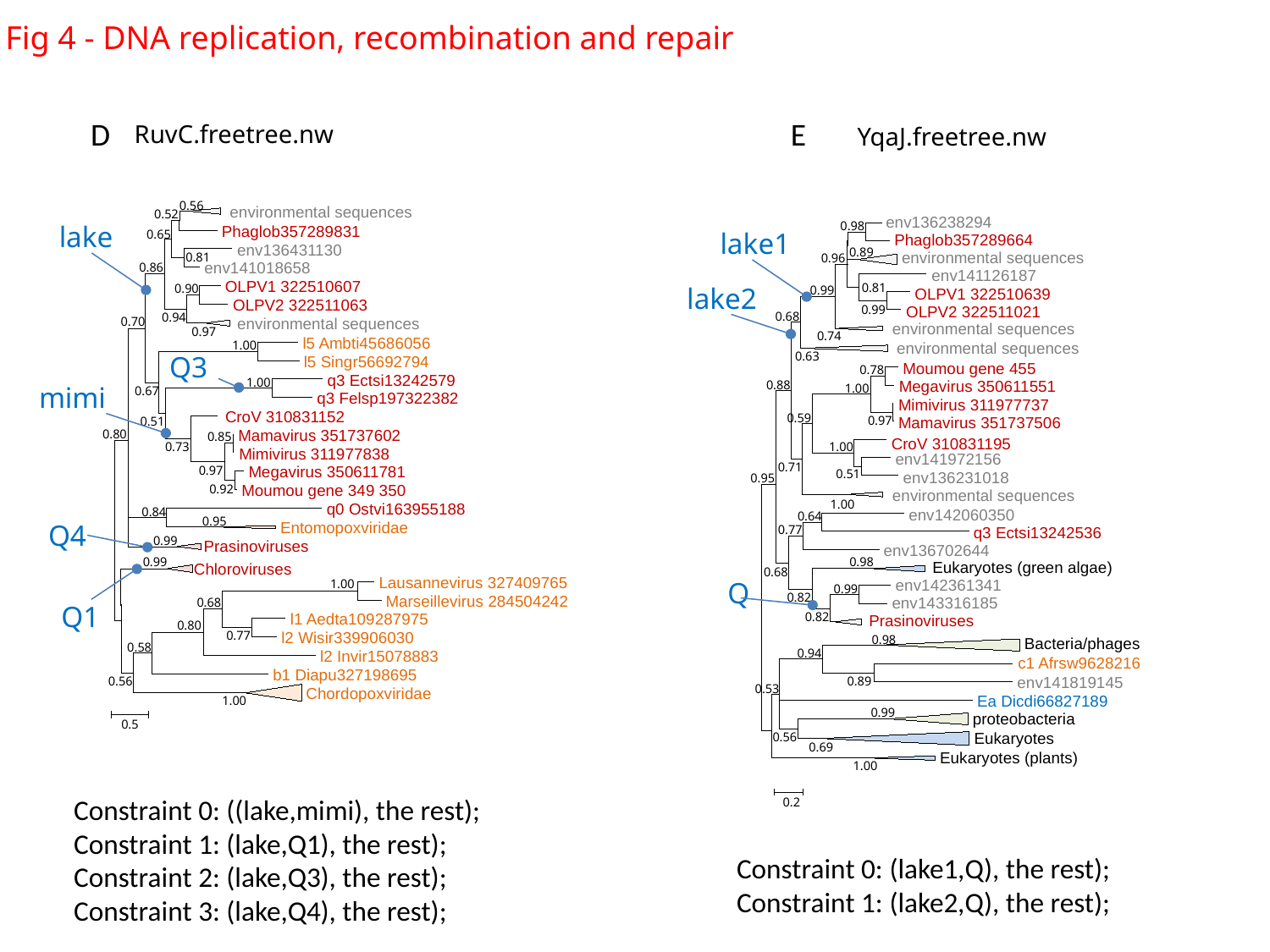

Fig 4 - DNA replication, recombination and repair
D
E
 YqaJ.freetree.nw
 RuvC.freetree.nw
0.56
 environmental sequences
0.52
 Phaglob357289831
0.65
 env136431130
0.81
 env141018658
0.86
 OLPV1 322510607
0.90
 OLPV2 322511063
0.94
 environmental sequences
0.70
0.97
 l5 Ambti45686056
1.00
 l5 Singr56692794
 q3 Ectsi13242579
1.00
0.67
 q3 Felsp197322382
 CroV 310831152
0.51
 Mamavirus 351737602
0.80
0.85
0.73
 Mimivirus 311977838
 Megavirus 350611781
0.97
 Moumou gene 349 350
0.92
 q0 Ostvi163955188
0.84
0.95
 Entomopoxviridae
0.99
Prasinoviruses
0.99
Chloroviruses
 Lausannevirus 327409765
1.00
 Marseillevirus 284504242
0.68
 l1 Aedta109287975
0.80
 l2 Wisir339906030
0.77
0.58
 l2 Invir15078883
 b1 Diapu327198695
0.56
 Chordopoxviridae
1.00
0.5
lake
Q3
mimi
Q4
Q1
 env136238294
0.98
 Phaglob357289664
0.89
 environmental sequences
0.96
 env141126187
0.81
0.99
 OLPV1 322510639
 OLPV2 322511021
0.99
0.68
 environmental sequences
0.74
 environmental sequences
0.63
 Moumou gene 455
0.78
 Megavirus 350611551
0.88
1.00
 Mimivirus 311977737
0.59
 Mamavirus 351737506
0.97
 CroV 310831195
1.00
 env141972156
0.71
0.51
 env136231018
0.95
 environmental sequences
1.00
 env142060350
0.64
0.77
 q3 Ectsi13242536
 env136702644
0.98
Eukaryotes (green algae)
 env142361341
0.99
0.82
 env143316185
0.82
Prasinoviruses
0.68
0.98
 Bacteria/phages
0.94
 c1 Afrsw9628216
 env141819145
0.89
0.53
 Ea Dicdi66827189
0.99
 proteobacteria
 Eukaryotes
0.56
0.69
 Eukaryotes (plants)
1.00
0.2
lake1
lake2
Q
Constraint 0: ((lake,mimi), the rest);
Constraint 1: (lake,Q1), the rest);
Constraint 2: (lake,Q3), the rest);
Constraint 3: (lake,Q4), the rest);
Constraint 0: (lake1,Q), the rest);
Constraint 1: (lake2,Q), the rest);

## Slide 5
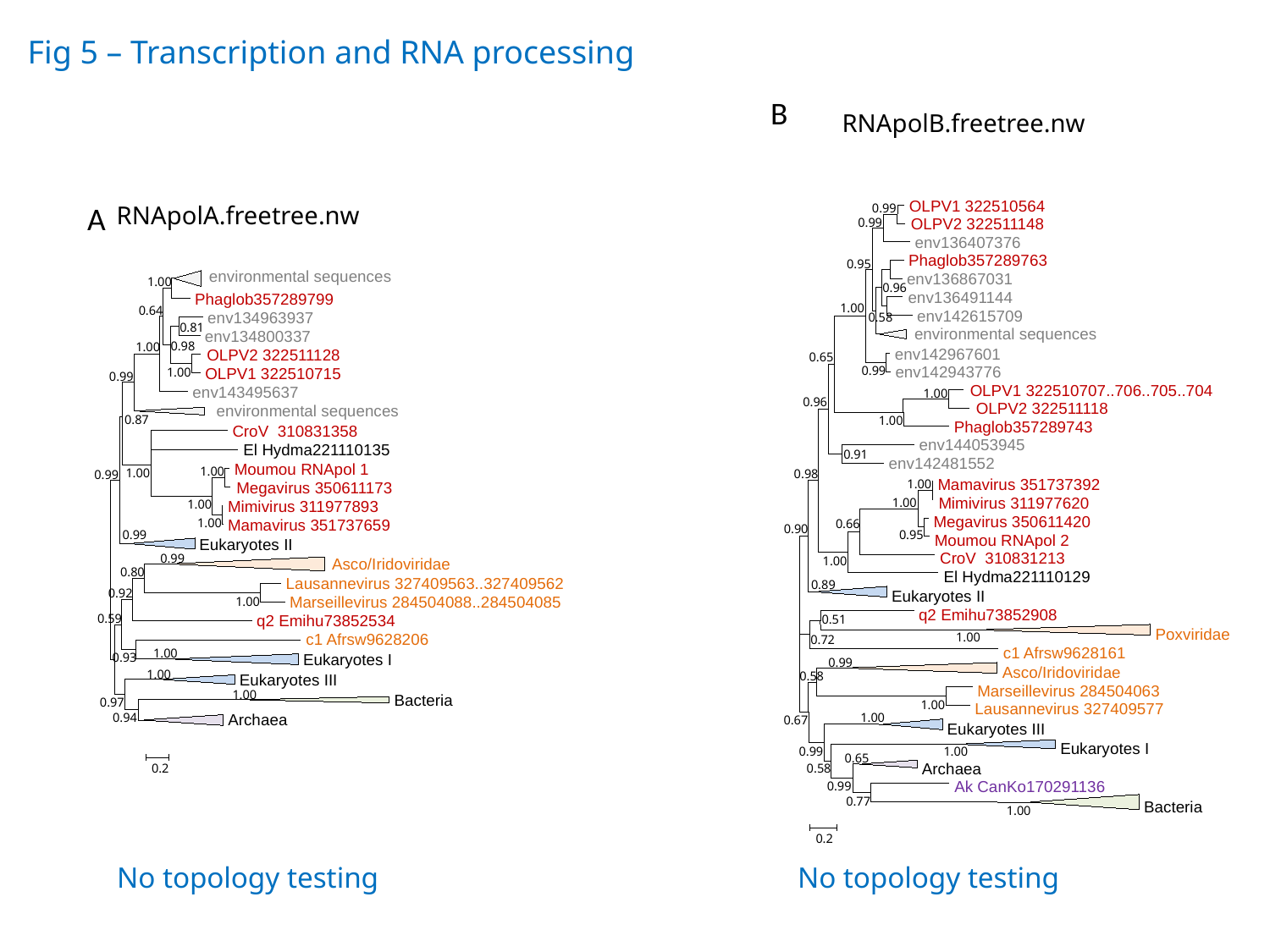

Fig 5 – Transcription and RNA processing
B
RNApolB.freetree.nw
A
 OLPV1 322510564
0.99
 OLPV2 322511148
0.99
 env136407376
 Phaglob357289763
0.95
 env136867031
0.96
 env136491144
1.00
 env142615709
0.58
 environmental sequences
 env142967601
0.65
 env142943776
0.99
 OLPV1 322510707..706..705..704
1.00
0.96
 OLPV2 322511118
1.00
 Phaglob357289743
 env144053945
0.91
 env142481552
0.98
 Mamavirus 351737392
1.00
 Mimivirus 311977620
1.00
 Megavirus 350611420
0.66
0.90
0.95
 Moumou RNApol 2
 CroV 310831213
1.00
 El Hydma221110129
0.89
 Eukaryotes II
 q2 Emihu73852908
0.51
 Poxviridae
1.00
0.72
 c1 Afrsw9628161
0.99
 Asco/Iridoviridae
0.58
 Marseillevirus 284504063
1.00
 Lausannevirus 327409577
1.00
0.67
 Eukaryotes III
 Eukaryotes I
1.00
0.99
0.65
 Archaea
0.58
 Ak CanKo170291136
0.99
0.77
 Bacteria
1.00
0.2
RNApolA.freetree.nw
 environmental sequences
1.00
 Phaglob357289799
0.64
 env134963937
0.81
 env134800337
0.98
1.00
 OLPV2 322511128
 OLPV1 322510715
1.00
0.99
 env143495637
 environmental sequences
0.87
 CroV 310831358
 El Hydma221110135
 Moumou RNApol 1
1.00
1.00
0.99
 Megavirus 350611173
 Mimivirus 311977893
1.00
 Mamavirus 351737659
1.00
0.99
 Eukaryotes II
0.99
 Asco/Iridoviridae
0.80
 Lausannevirus 327409563..327409562
0.92
 Marseillevirus 284504088..284504085
1.00
0.59
 q2 Emihu73852534
 c1 Afrsw9628206
1.00
 Eukaryotes I
0.93
1.00
 Eukaryotes III
1.00
 Bacteria
0.97
0.94
 Archaea
0.2
No topology testing
No topology testing

## Slide 6
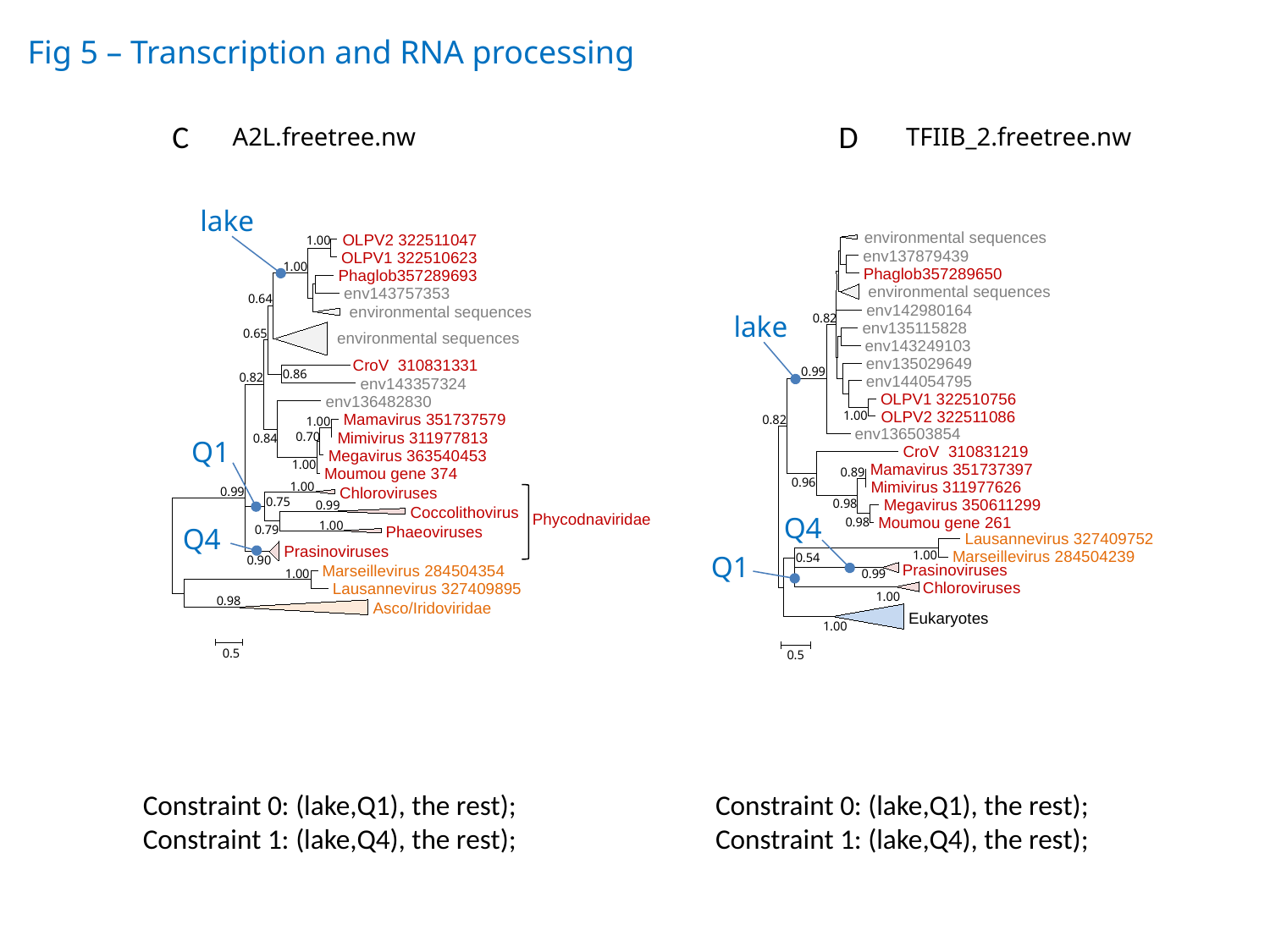

Fig 5 – Transcription and RNA processing
C
D
A2L.freetree.nw
 TFIIB_2.freetree.nw
lake
 environmental sequences
 env137879439
 Phaglob357289650
 environmental sequences
 env142980164
0.82
 env135115828
 env143249103
 env135029649
0.99
 env144054795
 OLPV1 322510756
 OLPV2 322511086
1.00
0.82
 env136503854
 CroV 310831219
 Mamavirus 351737397
0.89
0.96
 Lausannevirus 327409752
 Marseillevirus 284504239
1.00
0.54
 Prasinoviruses
0.99
 Chloroviruses
1.00
 Eukaryotes
1.00
 Mimivirus 311977626
 Megavirus 350611299
0.98
 Moumou gene 261
0.98
0.5
 OLPV2 322511047
1.00
 OLPV1 322510623
1.00
 Phaglob357289693
 env143757353
0.64
 environmental sequences
0.65
 environmental sequences
CroV 310831331
0.86
0.82
 env143357324
 env136482830
 Mamavirus 351737579
1.00
 Mimivirus 311977813
0.70
0.84
 Megavirus 363540453
1.00
 Moumou gene 374
1.00
 Chloroviruses
0.99
0.75
0.99
 Coccolithovirus
 Phycodnaviridae
1.00
0.79
 Phaeoviruses
 Prasinoviruses
0.90
Marseillevirus 284504354
1.00
Lausannevirus 327409895
0.98
 Asco/Iridoviridae
0.5
lake
Q1
Q4
Q4
Q1
Constraint 0: (lake,Q1), the rest);
Constraint 1: (lake,Q4), the rest);
Constraint 0: (lake,Q1), the rest);
Constraint 1: (lake,Q4), the rest);

## Slide 7
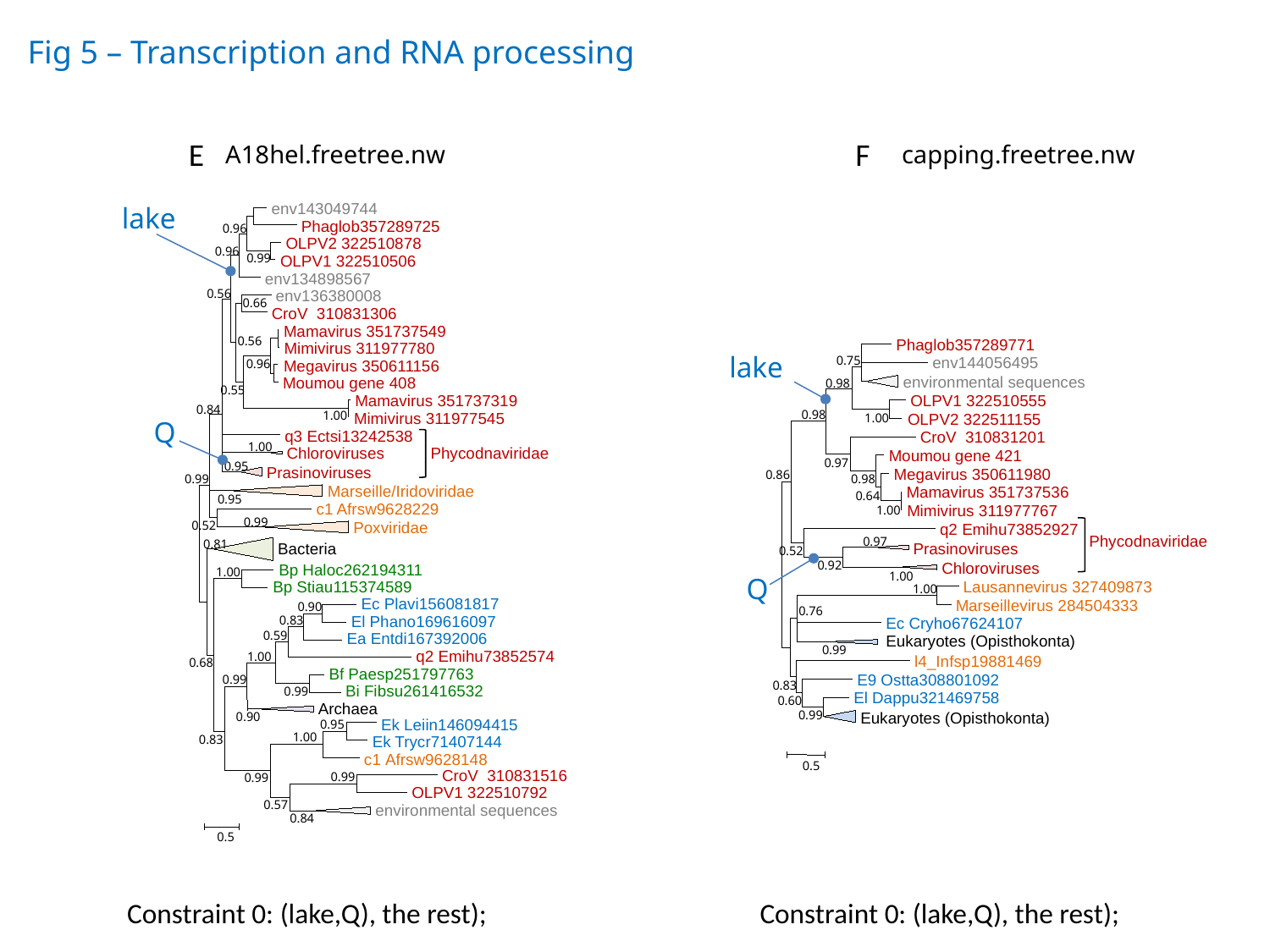

Fig 5 – Transcription and RNA processing
E
F
 capping.freetree.nw
 A18hel.freetree.nw
 env143049744
 Phaglob357289725
0.96
 OLPV2 322510878
0.96
0.99
 OLPV1 322510506
 env134898567
 env136380008
0.56
0.66
 CroV 310831306
 Mamavirus 351737549
0.56
 Mimivirus 311977780
 Megavirus 350611156
0.96
 Moumou gene 408
0.55
 Mamavirus 351737319
0.84
1.00
 Mimivirus 311977545
 q3 Ectsi13242538
1.00
 Phycodnaviridae
 Chloroviruses
0.95
 Prasinoviruses
0.99
 Marseille/Iridoviridae
0.95
 c1 Afrsw9628229
0.99
 Poxviridae
0.52
0.81
 Bacteria
 Bp Haloc262194311
1.00
 Bp Stiau115374589
 Ec Plavi156081817
0.90
 El Phano169616097
0.83
0.59
 Ea Entdi167392006
 q2 Emihu73852574
1.00
0.68
 Bf Paesp251797763
0.99
 Bi Fibsu261416532
0.99
 Archaea
0.90
 Ek Leiin146094415
0.95
1.00
 Ek Trycr71407144
 c1 Afrsw9628148
 CroV 310831516
0.99
0.99
 OLPV1 322510792
0.57
 environmental sequences
0.84
0.83
0.5
lake
Q
 Phaglob357289771
 env144056495
0.75
 environmental sequences
0.98
 OLPV1 322510555
0.98
 OLPV2 322511155
1.00
 CroV 310831201
 Moumou gene 421
0.97
 Megavirus 350611980
0.86
0.98
 Mamavirus 351737536
0.64
 Mimivirus 311977767
1.00
 q2 Emihu73852927
0.97
 Prasinoviruses
0.52
0.92
 Chloroviruses
1.00
 Lausannevirus 327409873
1.00
 Marseillevirus 284504333
0.76
 Ec Cryho67624107
 Eukaryotes (Opisthokonta)
0.99
 l4_Infsp19881469
 E9 Ostta308801092
0.83
 El Dappu321469758
0.60
0.99
 Eukaryotes (Opisthokonta)
0.5
 Phycodnaviridae
lake
Q
Constraint 0: (lake,Q), the rest);
Constraint 0: (lake,Q), the rest);

## Slide 8
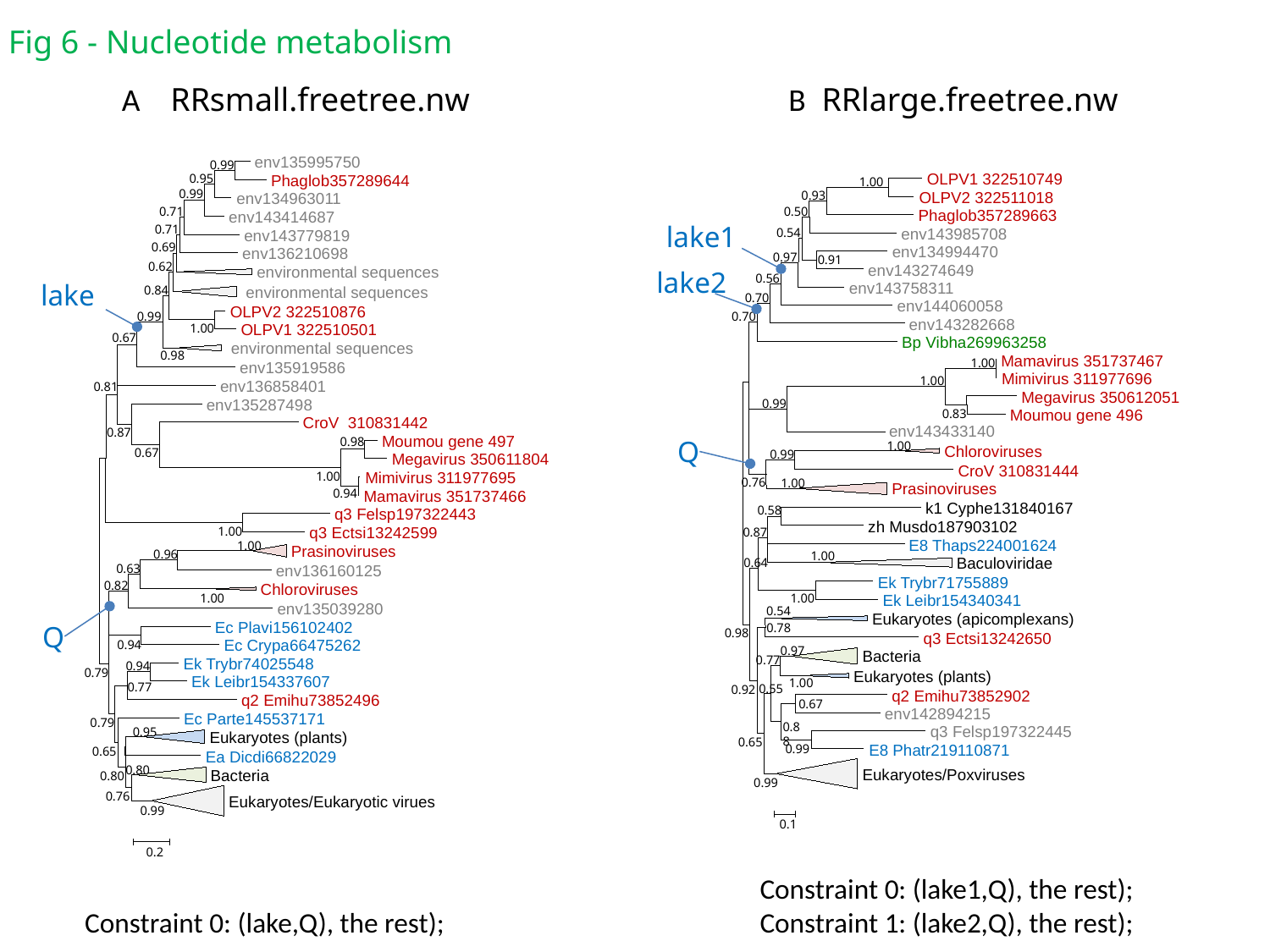

Fig 6 - Nucleotide metabolism
A
B
 RRsmall.freetree.nw
 RRlarge.freetree.nw
 env135995750
0.99
 OLPV1 322510749
1.00
 OLPV2 322511018
0.93
0.50
 Phaglob357289663
 env143985708
0.54
 env134994470
0.97
0.91
 env143274649
0.56
 env143758311
0.70
 env144060058
0.70
 env143282668
 Bp Vibha269963258
 Mamavirus 351737467
1.00
 Mimivirus 311977696
1.00
 Megavirus 350612051
0.99
 Moumou gene 496
0.83
 env143433140
1.00
 Chloroviruses
0.99
 CroV 310831444
0.76
1.00
 Prasinoviruses
 k1 Cyphe131840167
0.58
 zh Musdo187903102
0.87
 E8 Thaps224001624
1.00
 Baculoviridae
0.64
 Ek Trybr71755889
1.00
 Ek Leibr154340341
0.54
 Eukaryotes (apicomplexans)
0.78
0.98
 q3 Ectsi13242650
0.97
 Bacteria
0.77
 Eukaryotes (plants)
1.00
0.55
0.92
 q2 Emihu73852902
0.67
 env142894215
0.88
 q3 Felsp197322445
0.65
 E8 Phatr219110871
0.99
 Eukaryotes/Poxviruses
0.99
0.1
 Phaglob357289644
0.95
0.99
 env134963011
0.71
 env143414687
lake1
0.71
 env143779819
0.69
 env136210698
0.62
 environmental sequences
lake2
lake
 environmental sequences
0.84
 OLPV2 322510876
0.99
 OLPV1 322510501
1.00
0.67
 environmental sequences
0.98
 env135919586
 env136858401
0.81
 env135287498
 CroV 310831442
0.87
 Moumou gene 497
0.98
Q
0.67
 Megavirus 350611804
 Mimivirus 311977695
1.00
 Mamavirus 351737466
0.94
 q3 Felsp197322443
 q3 Ectsi13242599
1.00
1.00
 Prasinoviruses
0.96
 env136160125
0.63
0.82
 Chloroviruses
1.00
 env135039280
Q
 Ec Plavi156102402
 Ec Crypa66475262
0.94
 Ek Trybr74025548
0.94
0.79
 Ek Leibr154337607
0.77
 q2 Emihu73852496
 Ec Parte145537171
0.79
0.95
 Eukaryotes (plants)
0.65
 Ea Dicdi66822029
0.80
 Bacteria
0.80
0.76
 Eukaryotes/Eukaryotic virues
0.99
0.2
Constraint 0: (lake1,Q), the rest);
Constraint 1: (lake2,Q), the rest);
Constraint 0: (lake,Q), the rest);

## Slide 9
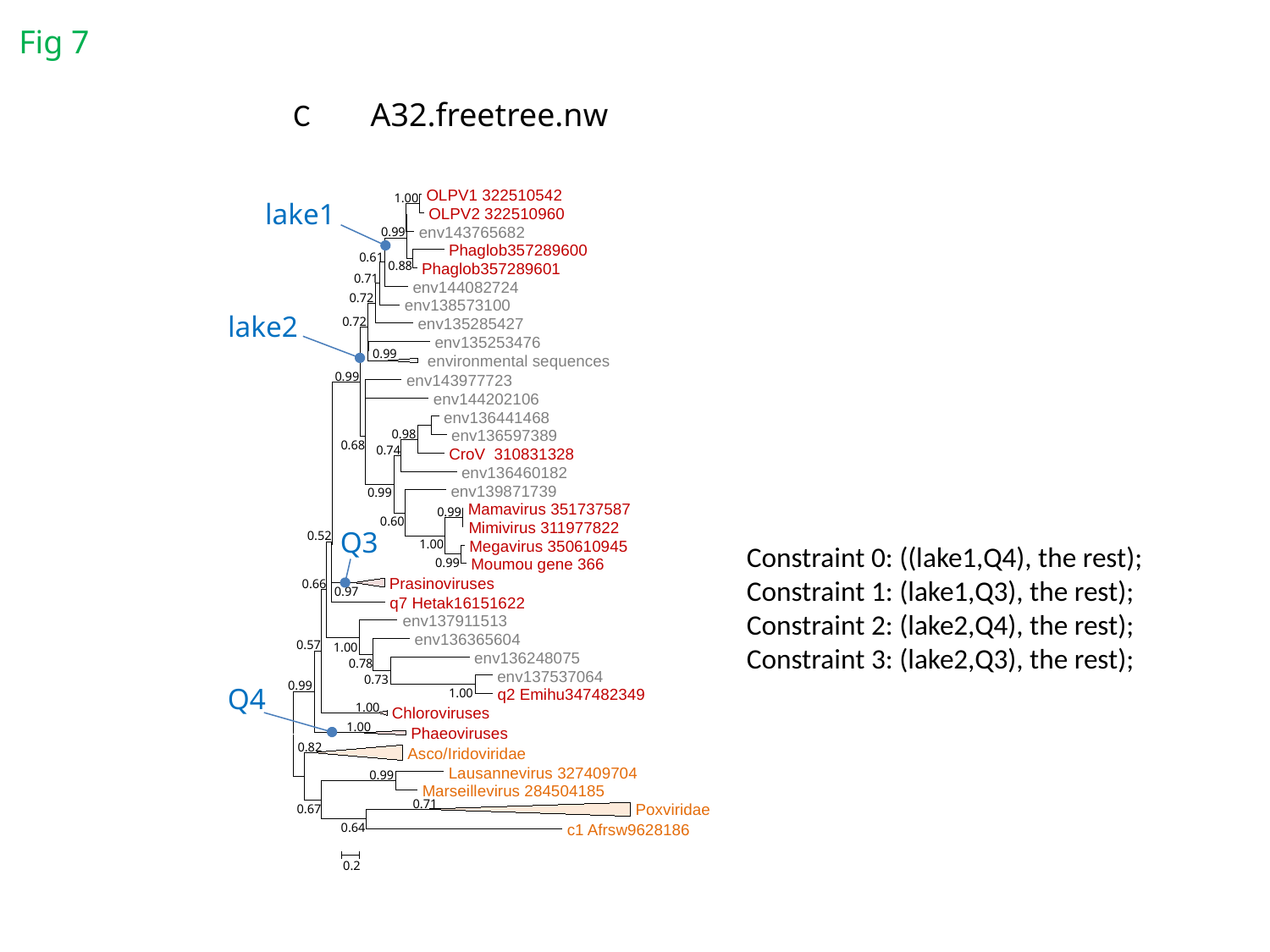

Fig 7
C
 A32.freetree.nw
 OLPV1 322510542
1.00
 OLPV2 322510960
 env143765682
0.99
 Phaglob357289600
0.61
0.88
 Phaglob357289601
0.71
 env144082724
0.72
 env138573100
 env135285427
0.72
 env135253476
0.99
 environmental sequences
0.99
 env143977723
 env144202106
 env136441468
 env136597389
0.98
0.68
0.74
 CroV 310831328
 env136460182
 env139871739
0.99
 Mamavirus 351737587
0.99
0.60
 Mimivirus 311977822
0.52
 Megavirus 350610945
1.00
 Moumou gene 366
0.99
 Prasinoviruses
0.66
0.97
 q7 Hetak16151622
 env137911513
 env136365604
0.57
1.00
 env136248075
0.78
 env137537064
0.73
0.99
 q2 Emihu347482349
1.00
1.00
 Chloroviruses
1.00
 Phaeoviruses
0.82
 Asco/Iridoviridae
 Lausannevirus 327409704
0.99
 Marseillevirus 284504185
0.71
 Poxviridae
0.67
 c1 Afrsw9628186
0.64
0.2
lake1
lake2
Q3
Constraint 0: ((lake1,Q4), the rest);
Constraint 1: (lake1,Q3), the rest);
Constraint 2: (lake2,Q4), the rest);
Constraint 3: (lake2,Q3), the rest);
Q4

## Slide 10
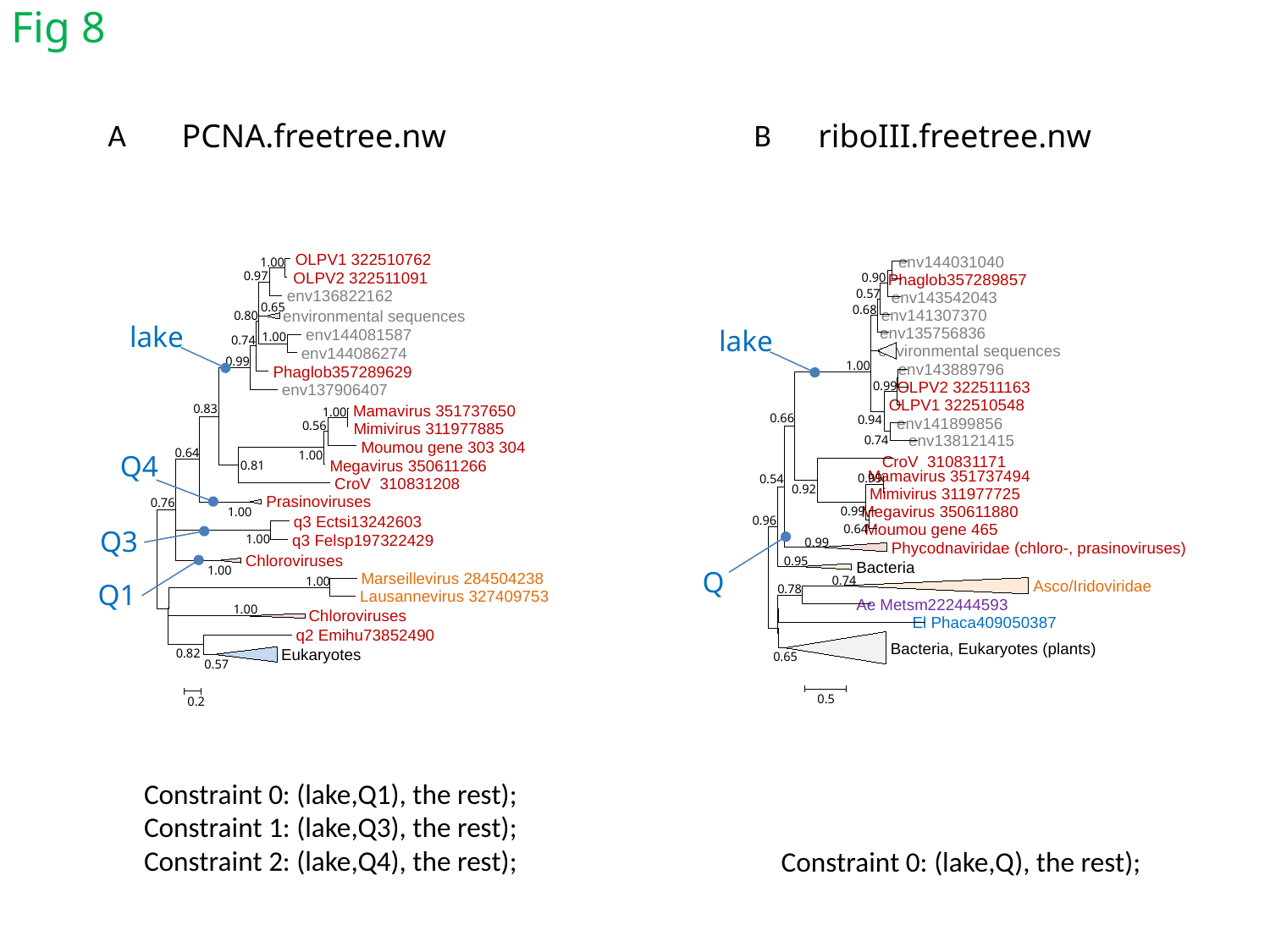

Fig 8
PCNA.freetree.nw
# riboIII.freetree.nw
A
B
 OLPV1 322510762
1.00
 OLPV2 322511091
0.97
 env136822162
0.65
 environmental sequences
0.80
 env144081587
1.00
0.74
 env144086274
0.99
 Phaglob357289629
 env137906407
 Mamavirus 351737650
0.83
1.00
0.56
 Mimivirus 311977885
 Moumou gene 303 304
0.64
1.00
 Megavirus 350611266
0.81
 CroV 310831208
 Prasinoviruses
0.76
1.00
 q3 Ectsi13242603
 q3 Felsp197322429
1.00
 Chloroviruses
1.00
 Marseillevirus 284504238
1.00
 Lausannevirus 327409753
1.00
 Chloroviruses
 q2 Emihu73852490
 Eukaryotes
0.82
0.57
0.2
 env144031040
 Phaglob357289857
0.90
0.57
 env143542043
0.68
 env141307370
 env135756836
 environmental sequences
1.00
 env143889796
 OLPV2 322511163
0.99
 OLPV1 322510548
0.66
0.94
 env141899856
 env138121415
0.74
 CroV 310831171
 Mamavirus 351737494
0.99
0.54
0.92
 Mimivirus 311977725
 Megavirus 350611880
0.99
0.96
 Moumou gene 465
0.64
0.99
 Phycodnaviridae (chloro-, prasinoviruses)
0.95
 Bacteria
0.74
 Asco/Iridoviridae
0.78
 Ae Metsm222444593
 El Phaca409050387
 Bacteria, Eukaryotes (plants)
0.65
0.5
lake
lake
Q4
Q3
Q
Q1
Constraint 0: (lake,Q1), the rest);
Constraint 1: (lake,Q3), the rest);
Constraint 2: (lake,Q4), the rest);
Constraint 0: (lake,Q), the rest);
